# Mechanisms of vaccine protection in chickens against challenge with virulent *Mycoplasma synoviae*

**DOI:** 10.1186/s13567-025-01571-3

**Published:** 2025-07-09

**Authors:** Kanishka I. Kamathewatta, Anna Kanci Condello, Pollob K. Shil, Amir H. Noormohammadi, Neil D. Young, Glenn F. Browning, Kelly A. Tivendale, Nadeeka K. Wawegama

**Affiliations:** 1 Asia-Pacific Centre for Animal Health, Melbourne Veterinary School, Faculty of Science, The University of Melbourne, Parkville, VIC Australia; 2 Asia-Pacific Centre for Animal Health, Melbourne Veterinary School, Faculty of Science, The University of Melbourne, Werribee, VIC Australia; 3 Department of Veterinary Biosciences, Melbourne Veterinary School, Faculty of Science, The University of Melbourne, Parkville, VIC Australia

**Keywords:** Vaxsafe^®^ MS, *Mycoplasma synoviae*, chicken tracheal mucosae, vaccine protection, RNA sequencing

## Abstract

The Vaxsafe MS vaccine is used globally to control infections with *Mycoplasma synoviae* (MS) in commercial poultry. It provides long-term protective immunity against airsacculitis and tracheitis caused by *M. synoviae*. However, the mechanisms involved in the protection afforded by the vaccine are not well understood. This study aimed to investigate and compare tracheal mucosal responses to challenge with virulent *M. synoviae* in chickens vaccinated with Vaxsafe MS and in unvaccinated chickens. The tracheal mucosal transcriptional profiles were obtained using messenger RNA sequencing. Compared to the unvaccinated-unchallenged chickens, 64 genes were differentially transcribed in the vaccinated-challenged chickens and 321 genes were differentially transcribed in the unvaccinated-challenged chickens. In vaccinated-challenged chickens, functional categories enriched with up-regulated genes included IL4 production, TCR signalling (without T-cell co-stimulation), T-cell activation/proliferation/differentiation and B-cell activation/proliferation. In the unvaccinated-challenged chickens, many other functional categories were also enriched with upregulated genes, including Positive regulation of the MAPK/ERK cascades, Positive regulation of IFN gamma production, T-cell co-stimulation, Negative regulation of IL6 production, IL10 production, Toll-like receptor signalling, Neutrophil activation/degranulation, DAP12 signalling, Chemotaxis, Phagocytosis, Cell killing, PD-1 signalling, Negative regulation of non-canonical NF-κB signal transduction, Isotype switching and Somatic diversification of immune receptors. The transcriptional changes in the unvaccinated-challenged chickens were indicative of T-helper-1-cell-mediated inflammation and dysregulated primary T- and B-cell responses, which were absent in the vaccinated-challenged chickens. In the vaccinated-challenged chickens the changes were indicative of a memory T-follicular-helper-cell-dependent secondary B-cell response, suggesting that this response was protective against the dysregulated inflammatory response seen in unvaccinated chickens.

**Supplementary Information:**

The online version contains supplementary material available at 10.1186/s13567-025-01571-3.

## Introduction

*Mycoplasma synoviae* (MS) causes infectious synovitis and respiratory tract infection in chickens. The respiratory tract infection is usually mild or sub-clinical [1], but the severity is markedly enhanced by concurrent infections with infectious bronchitis virus (IBV), Newcastle disease virus and *Escherichia coli* [2, 3]. Therefore, MS and IBV co-infection models are commonly used to study the respiratory tract infection caused by MS [2–4]. In a previous study, we identified immunopathological transcriptional changes in the chicken trachea in response to chronic MS infection, using a MS-IBV co-infection model [5].

Control of disease caused by infection with MS in commercial poultry is achieved by maintaining MS-free breeder flocks, medication and vaccination [1]. The live-attenuated Vaxsafe MS vaccine (MS-H strain) is used globally to control MS infections in commercial poultry. It was created by chemical mutagenesis of the Australian MS field isolate 86079/7NS and selection based on temperature sensitivity [6]. It is safe and effective in chickens and turkeys [7, 8] and provides long term protection against respiratory tract lesions induced by infection with virulent MS [9]. The MS-H vaccine has been shown to induce detectable serum agglutinating antibody by three weeks after vaccination and to provide full protective immunity against challenge with virulent MS from four weeks after vaccination [10].

The host responses and mechanisms involved in the protection offered by the Vaxsafe MS vaccine are not completely understood. A previous study has delineated the mechanisms involved in the primary immune response induced by the MS-H strain, based on indirect immunofluorescent staining of tracheal mucosal cellular infiltrates and measurements of the levels of mRNA transcripts for 8 cytokines [11]. Experimental inoculation of chickens with MS-H induced a T_H_17 response while inoculation with the parental strain 86079/7NS induced a T_H_1 response. This study further highlighted a lack of T-cell mediated immunity and early B-cell recruitment in response to vaccination with MS-H [11]. A further study demonstrated a T_H_1 cytotoxic cell-mediated response in the tracheal mucosa of chickens vaccinated with Vaxsafe MS at 7 and 21 days after challenge with virulent *M. synoviae* [12]. However, this study employed a targeted approach and focused only on differential transcription of 6 cytokine genes and immunofluorescent staining of 6 immune cell types. Detailed exploration of other transcriptional changes in the trachea following infection with *M. synoviae* in Vaxsafe MS-vaccinated chickens would provide a more comprehensive understanding of vaccine-mediated immune protection against *M. synoviae*.

The study described here aimed to explore global tracheal mucosal transcriptional changes in response to challenge with the virulent Australian MS field strain 94011 V-18d in Vaxsafe MS-vaccinated chickens and to compare them to those of unvaccinated chickens. This would allow identification of the protective immune mechanisms induced by the vaccine and its role in preventing the immunopathological damage caused by virulent MS.

## Materials and methods

### Experimental vaccination and infection of chickens

Specific-pathogen-free (SPF) eggs supplied by Australian SPF Services Pty Ltd (Woodend, Victoria, Australia) were hatched and raised in microbiologically secure isolators at the Asia–Pacific Centre for Animal Health (APCAH) at the Melbourne Veterinary School (Werribee, Victoria, Australia). Thirty birds were randomly assigned into three groups of ten, and each group was placed in a separate microbiologically secure isolator. Ten 4-week-old SPF chickens were vaccinated by eye drop with 0.033 mL of Vaxsafe MS/bird, a dose of 10^5.7^ colour-changing units (CCU) of the live-attenuated vaccine. At 8 weeks of age, they were challenged concurrently with IBV and MS, by intratracheal inoculation of 10^2.0^ median egg infectious doses (EID_50_) of the Australian IBV field strain V1/71/bird and exposure to an aerosol of 10^7.0^ CCU of the Australian MS field strain 94011 V-18d/mL. This group served as the vaccinated-challenged group. Another group of 10 chickens were inoculated by eye drop with 0.033 mL of MS broth/bird at 4 weeks of age and then challenged at 8 weeks of age concurrently with IBV and MS, by intratracheal inoculation with 10^2.0^ EID_50_ of IBV strain V1/71/bird and exposure to an aerosol of 10^7.0^ CCU of MS strain 94011 V-18d/mL. This group served as the unvaccinated-challenged positive control group. The intratracheal inoculation of the IBV strain was performed using an 18-gauge Vetafarm medication needle, as described previously [10]. Aerosol delivery of the *M. synoviae* field strain was performed in a purpose-built infection chamber as described previously [13]. Another group of 10 chickens were inoculated at 4 weeks of age by eye drop with 0.033 mL of MS broth/bird and remained unchallenged. This group served as the unvaccinated-unchallenged negative control group. All birds were humanely euthanised and necropsied at 10 weeks of age (2 weeks after the challenge).

### Histopathological assessment of tracheal mucosal thicknesses

Samples of the upper trachea from each bird were fixed in 10% neutral-buffered formalin for at least 24 h and processed for histological sectioning. Cross Sects. (5 µm) sliced from these samples were stained with hematoxylin and eosin (H&E). Six measurements of tracheal mucosal thicknesses were made using a light microscope at × 400 magnification. The mean tracheal mucosal thicknesses of the upper trachea of the three experimental groups were compared using an ordinary one-way ANOVA, and Tukey’s multiple comparisons tests, in GraphPad Prism version 9.3.1 for Windows (GraphPad Software, La Jolla, CA, USA).

### Extraction of total RNA

Tracheal samples collected and stabilised in RNAlater solution (Invitrogen, Carlsbad, CA, USA) during the necropsies were stored at −20 °C until further processed. Tracheal mucosae were separated as described previously [14] from all birds (10) in the vaccinated-challenged and unvaccinated-challenged groups and from 4 birds in the unvaccinated-unchallenged group. Approximately 20 mg of tracheal mucosal tissue was used as the starting material and total RNA was extracted using the RNeasy Mini kit (QIAGEN, Hilden, Germany) according to the manufacturer’s recommendations. DNA contamination was removed using a TURBO DNA-free kit (Invitrogen, Carlsbad, CA, USA) using the rigorous DNase treatment protocol with two-step incubation. Finally, RNA extracts were cleaned and concentrated using a Zymo RNA Clean & Concentrator-25 kit (Zymo Research Corporation, Irvine, CA, USA) according to the manufacturer’s recommendations. The RNA concentration was measured using a Qubit 4 fluorometer (Thermo Fisher Scientific, Waltham, MA, USA). RNA integrity was confirmed using the Agilent 4200 TapeStation system using an RNA ScreenTape assay (Agilent Technologies, Santa Clara, CA, USA). Two samples from the vaccinated-challenged group were removed from the study because of the poor integrity of the extracted RNA.

### RNA sequencing

The TruSeq Stranded mRNA library preparation kit and protocol (Illumina Inc., San Diego, CA, USA) were used to prepare mRNA sequencing libraries. Briefly, mRNA was purified from approximately 500 ng of total RNA and fragmented mRNA was used to synthesise cDNA. The cDNA was end-repaired and adapter-ligated before enriching to obtain a sequencing library of 1 nM to 100 nM molarity with a majority of DNA fragments distributed between 200 and 600 base pairs. The Agilent 4200 TapeStation system (Agilent Technologies, Santa Clara, CA, USA) was used to evaluate the fragment size, fragment distribution and molarity of the cDNA libraries. The libraries were pooled by normalising to 1 nM and were sequenced on a NextSeq500 sequencing platform (Illumina Inc., San Diego, CA, USA) at the Walter and Eliza Hall Institute of Medical Research (Melbourne, Victoria, Australia), to obtain 166 bp paired-end reads.

### Assessment and quality control of RNA sequencing data

FastQC [15] version 0.11.9 was used to assess the quantity and quality of RNA sequence reads. Illumina adapters were trimmed and base calls with PHRED quality scores ≤ 20 were removed using Trim Galore version 0.6.7 [16]. Paired reads were checked for singletons and they were removed using repair.sh in BBMap version 38.96 [17].

### Calculation of gene transcription

The *Gallus gallus* (chicken) genome was downloaded in fasta and gtf formats from the Ensembl database release 106 [18]. A reference database was prepared from the mRNA sequences of protein-coding genes using RSEM version 1.3.3 [19]. RSEM version 1.3.3 used Bowtie2 version 2.4.5 [20] to map paired RNA-seq reads to the reference database. Expected read counts per gene were determined using RSEM version 1.3.3.

### Differential gene transcription analysis

Differential gene transcription analysis was performed in RStudio Version 1.4.1103 according to published guidelines [21]. The raw read count data were transformed to counts-per-million (CPM) values using edgeR version 3.32.1 [22] to normalise for differences in library sizes. The genes with low CPM values in at least three samples were filtered out using the default cut-off in the “filterByExpr” function in edgeR version 3.32.1. The trimmed mean of M-values (TMM) method [23] was used to normalise gene transcription distributions using edgeR version 3.32.1. The package limma version 3.46.0 [24] was used to remove heteroscedascity from count data using the voomWithQualityWeights function [25] and to log_2_ transform CPM values. Linear modelling in limma version 3.46.0 was performed on log_2_ CPM values. Genes with a fold change ≥ 2 at a false discovery rate (FDR) < 0.05 were considered to be significantly differentially transcribed.

### Gene set enrichment analysis

The package topGO version 2.42.0 [26] was used with the *G. gallus* gene ontology (GO) universe prepared as described previously [14] to identify enriched GO terms. The GO terms in the molecular functions (MFs), biological processes (BPs) and cellular components (CCs) categories were considered significantly enriched with differentially regulated genes at a FDR of < 0.05. The Reactome database version 77 [27] in the PANTHER online classification tool [28] was used to identify enriched biological pathways at a FDR of < 0.05.

## Results

### Unvaccinated-challenged chickens had significantly increased tracheal mucosal thicknesses compared to vaccinated-challenged chickens

**Figure 1:**
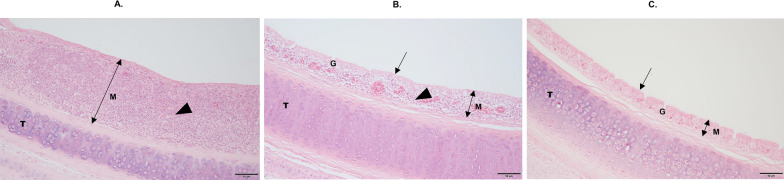
**Haematoxylin and eosin-stained upper tracheal cross sections.** **A** Unvaccinated-challenged group. **B** Vaccinated-challenged group. **C** Unvaccinated-unchallenged group. M, mucosa; T, tracheal cartilage; G, mucus glands; double-headed black arrows indicate the mucosal thickness; single-headed black arrows indicate ciliated pseudostratified columnar epithelia; black arrowheads indicate immune cell infiltration. The scale bars indicate 50 µm.

The mean upper tracheal mucosal thicknesses (TMTs) of unvaccinated-challenged, vaccinated-challenged and unvaccinated-unchallenged chickens were 142.5 ± 14.7 µm, 91.9 ± 6.0 µm and 68.4 ± 5.8 µm, respectively. The mean upper TMT of unvaccinated-challenged chickens was significantly greater than that of the vaccinated-challenged (*p* < 0.0043) and unvaccinated-unchallenged (*p* < 0.0001) chickens. The mean upper TMT of vaccinated-challenged chickens was not significantly different from that of the unvaccinated-unchallenged chickens. The increased TMTs in the unvaccinated-challenged chickens resulted from pronounced infiltration of immune cells, particularly lymphocytes and plasma cells, into the mucosa. The pseudostratified columnar epithelium and intraepithelial mucous glands were disrupted in these birds (Figure 1A). In vaccinated-challenged chickens, the tracheal mucosae were slightly thickened, with some infiltration of lymphocytes and plasma cells, and the ciliated pseudostratified columnar epithelium and intraepithelial mucous glands were intact (Figure 1B). In unvaccinated-unchallenged chickens, the ciliated pseudostratified columnar epithelium and the intraepithelial mucous glands were intact, with no infiltration of immune cells or tracheal mucosal thickening (Figure 1C).

### Sixty-four genes were differentially transcribed in vaccinated-challenged chickens compared to unvaccinated-unchallenged chickens

**Figure 2:**
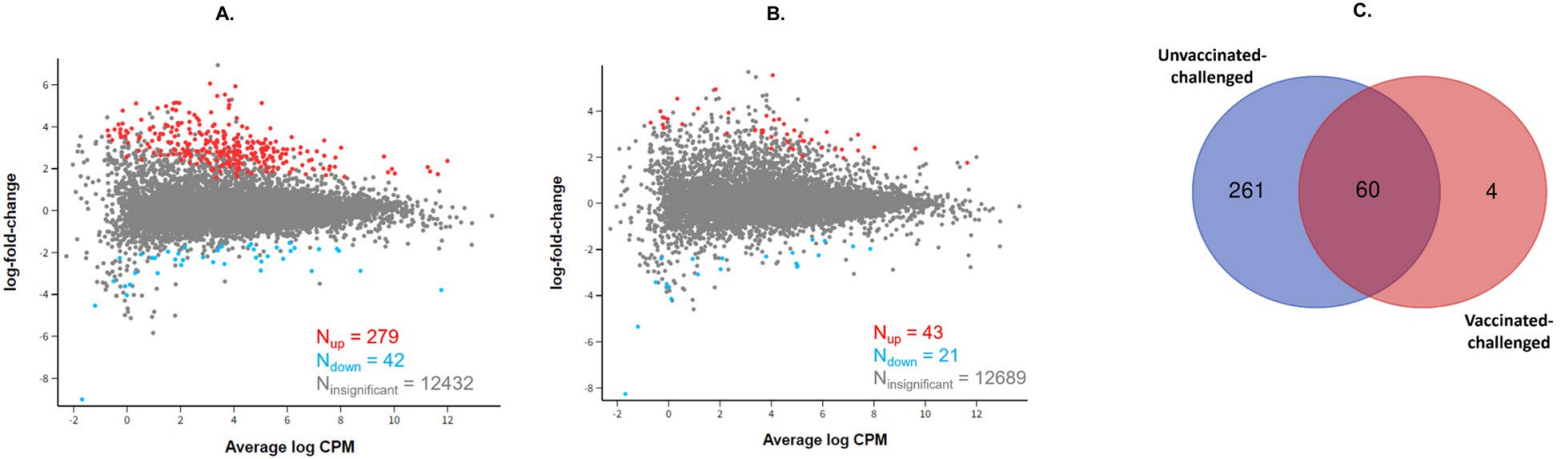
**Mean-difference plots of log fold change versus log CPM.** **A** Unvaccinated-challenged chickens compared to unvaccinated-unchallenged chickens. **B** Vaccinated-challenged chickens compared to unvaccinated-unchallenged chickens. N indicates the number of genes, red dots and numbers indicate up-regulated genes, blue dots and numbers indicate down-regulated genes, grey dots and numbers indicate the genes with no significant difference in transcription. **C** Venn diagram showing the differentially expressed genes that are unique to or shared by the two challenged groups of chickens. The blue circle represents the unvaccinated-challenged group (compared to unvaccinated-unchallenged birds) and the red circle represents the vaccinated-challenged group (compared to unvaccinated-unchallenged birds).

A total of 12 753 *G. gallus* protein coding genes were included in the differential gene transcription analysis. A total of 321 genes were differentially transcribed in the unvaccinated-challenged chickens compared to the unvaccinated-unchallenged negative control chickens. Among these, 279 were up-regulated and 42 genes were down-regulated (Figure 2A). In the vaccinated-challenged chickens only 64 genes were differentially transcribed, 43 of which were up-regulated and 21 of which were down-regulated (Figure 2B) compared to the unvaccinated-unchallenged negative control chickens. Sixty of these 64 genes were also differentially transcribed in the unvaccinated-challenged chickens compared to the unvaccinated-unchallenged negative control chickens. Only 4 genes were uniquely differentially transcribed in the vaccinated-challenged chickens compared to the unvaccinated-unchallenged negative control chickens (Figure 2C). There was no significant difference in gene transcription between the unvaccinated-challenged and the vaccinated-challenged chickens. Therefore, direct comparison between these two groups was not possible. Henceforth, differential transcription (up or down regulated genes) and functional enrichments are reported in the vaccinated-challenged or in the unvaccinated-challenged birds in comparison with the unvaccinated-unchallenged negative control group, unless otherwise stated.

### Genes that have immune modulatory roles were uniquely down-regulated in the vaccinated-challenged chickens

Three of the four genes that were uniquely differentially transcribed in the vaccinated-challenged chickens were down-regulated, while a single uniquely differentially transcribed gene was up-regulated (Table 1). The down-regulated genes were *PDK4*, *WNT9A* and *NR4A3*, which have roles in immune regulation. The *PDK4* gene is also implicated in the regulation of cellular metabolism. The single up-regulated gene has not been assigned a functional role.

**Table 1:** **Uniquely differentially transcribed genes in the vaccinated-challenged chickens compared to unvaccinated-unchallenged chickens**

Gene name	Gene product	LFC	FDR
*PDK4*	Pyruvate dehydrogenase kinase 4	−2.31	4.12E-02
*WNT9A*	Wnt family member 9A	−2.39	4.12E-02
*NR4A3*	Nuclear receptor subfamily 4 group A member 3	−2.61	4.12E-02
Unassigned	Uncharacterised protein	3.26	4.12E-02

LFC: log_2_ fold-change; FDR: false discovery rate (< 0.05 was considered significant).

### The most significantly up-regulated genes in the vaccinated-challenged chickens included T- and B-cell-response-associated genes

The top 20 most significantly up-regulated genes in the vaccinated-challenged chickens included 8 genes that have roles in T-cell-mediated responses and 3 genes that have roles in B-cell-mediated immune responses (Table 2). Genes involved in cell migration (2), lipid metabolism (2) and the acute phase response (1) were also among the most significantly up-regulated genes (Table 2).

**Table 2:** **Most significantly up-regulated genes in the vaccinated-challenged chickens compared to the unvaccinated-unchallenged chickens**

Gene name	Gene product	Vaccinated-challenged birds		Unvaccinated-challenged birds	
		LFC	FDR	LFC	FDR
T cell response					
* CD3D*	CD3d molecule	3.09	3.58E-03	3.51	4.78E-04
* IL16*	Interleukin 16	2.91	4.25E-03	3.22	5.64E-04
* EOMES*	Eomesodermin	4.94	5.21E-03	4.79	2.63E-03
* GNLY*	Granulysin	2.34	1.69E-02	2.74	5.77E-04
* CD3E*	CD3e molecule	1.96	2.16E-02	2.39	5.64E-04
* LCP1*	Lymphocyte cytosolic protein 1	2.43	2.57E-02	3.00	7.03E-04
* CARMIL2*	RGD motif, leucine rich repeats, tropomodulin domain and proline-rich containing	3.80	3.27E-02	4.10	4.55E-03
* PTPRC*	Protein tyrosine phosphatase, receptor type C	2.29	3.27E-02	2.62	2.13E-03
B cell response					
* CD72*	CD72 molecule	5.55	1.59E-02	5.93	2.13E-03
Unassigned	TNF_2 domain-containing protein	3.71	1.69E-02	4.16	1.62E-03
* TNFRSF13C*	TNF receptor superfamily member 13C	4.54	2.16E-02	5.12	2.13E-03
Cell migration					
LOC100857191	C–C motif chemokine 26-like	2.98	2.16E-02	3.35	2.13E-03
* DOCK2*	Dedicator of cytokinesis 2	2.70	3.27E-02	3.01	2.75E-03
Lipid metabolism					
* FADS6*	Fatty acid desaturase 6	3.73	1.69E-02	3.50	7.68E-03
* ABCA1*	ATP-binding cassette, sub-family A (ABC1), member 1	2.04	3.27E-02	1.85	2.85E-02
Acute phase response					
* LBP*	Lipopolysaccharide binding protein	2.37	2.16E-02	2.59	2.31E-03
Other					
* RAB44*	RAB44, member RAS oncogene family	2.88	2.16E-02	3.09	2.63E-03
* HAAO*	3-hydroxyanthranilate 3,4-dioxygenase	3.66	2.16E-02	3.81	3.91E-03
* CLEC5A*	C-type lectin domain family 5, member A	4.11	2.59E-02	4.89	1.71E-03
unassigned	Uncharacterised protein	3.41	2.59E-02	3.57	4.55E-03

LFC: log_2_ fold-change; FDR: false discovery rate (< 0.05 was considered significant).

### Differentially transcribed cytokine and cytokine receptor associated genes

Five genes that encode cytokines or chemokines were up-regulated in the unvaccinated-challenged chickens. In contrast, only the gene encoding IL16 was up-regulated in the vaccinated-challenged chickens. Another 12 genes that encode cytokine or chemokine receptors were up-regulated in the unvaccinated-challenged chickens. Of these, only the genes encoding BAFFR, IL2RG and IL21R were up-regulated in the vaccinated-challenged chickens. The gene encoding the chemokine CCL20 was down-regulated in both the unvaccinated-challenged and vaccinated-challenged chickens (Table 3).

**Table 3:** **Differentially transcribed cytokine/cytokine receptor encoding genes in the unvaccinated-challenged and/or vaccinated-challenged chickens, compared to unvaccinated-unchallenged chickens**

Ligand				Receptor			
Unvaccinated-challenged birds		Vaccinated-challenged birds		Unvaccinated-challenged birds		Vaccinated-challenged birds	
LFC	FDR	LFC	FDR	LFC	FDR	LFC	FDR
*TNFSF13B/BAFF*				*TNFRSF13C/BAFFR*			
3.27	6.83E-03			5.12	2.13E-03	4.54	2.16E-02
*TNFSF8*							
2.77	7.71E-03						
				*IL2RB*			
				1.79	2.88E-02		
				*IL2RG*			
				3.29	2.75E-03	2.76	4.95E-02
				*IL7R*			
				2.10	3.50E-02		
				*IL10RA*			
				1.99	2.77E-02		
				*IL12RB1*			
				3.86	6.52E-03		
*IL16*							
3.22	5.64E-04	2.91	4.25E-03				
				*IL20RA*			
				2.94	7.68E-03		
				*IL21R*			
				4.41	2.13E-03	3.61	4.12E-02
				*CSF2RB*			
				2.69	3.71E-02		
*CXCL13*							
5.54	2.08E-02						
				*C3AR1*			
				2.26	3.45E-02		
				*CCR2*			
				2.21	3.40E-02		
*CCL5*				*CCR5*			
2.43	6.83E-03			3.35	1.06E-02		
*CCL20*							
−2.86	5.10E-03	−2.75	3.39E-02				

LFC: log_2_ fold-change; FDR: false discovery rate (< 0.05 was considered significant).

### Other immune response-associated genes that were differentially transcribed

The genes encoding the CD3 delta (CD3D) and CD3 epsilon (CD3E) subunits of the T-cell receptor complex were up-regulated in the vaccinated-challenged chickens. In addition to these two genes, another 10 genes associated with immune cell receptors were up-regulated in the unvaccinated-challenged chickens (Table 4). Major histocompatibility complex (MHC) class I-and II-associated genes were also up-regulated in the unvaccinated-challenged chickens. None of these genes were differentially transcribed in the vaccinated-challenged chickens, except for the up-regulated MHC class I-associated gene *BF2* (Table 4). The toll-like receptor gene, *TLR1A*, was up-regulated only in the unvaccinated-challenged chickens (Table 4).

**Table 4:** **Differentially transcribed immune response associated genes in the unvaccinated-challenged and/or vaccinated-challenged chickens, compared to unvaccinated-unchallenged chickens**

Gene name	Unvaccinated-challenged birds		Vaccinated-challenged birds	
	LFC	FDR	LFC	FDR
Immune cell receptor-associated				
* FCRL4*	3.89	1.48E-02		
* IGLL1*	2.98	7.35E-03		
* CD79B*	3.92	9.13E-03		
* CD3D*	3.51	4.78E-04	3.09	3.58E-03
* CD3E*	2.38	5.64E-04	1.96	2.16E-02
* PDCD1LG2*	4.03	2.31E-03		
* CD28*	3.68	5.10E-03		
* CTLA4*	3.65	3.77E-02		
* CD48*	3.02	2.63E-03		
* LY9*	2.65	1.06E-02		
* CD247*	2.37	1.92E-02		
* CD8BP*	3.84	2.51E-02		
Major histocompatibility complex-associated				
* BLB1* (MHC II)	2.09	1.67E-02		
* BLB2* (MHC II)	1.99	1.56E-02		
* CD74* (MHC II)	2.08	3.02E-03		
* BF1* (MHC I)	1.83	5.60E-03		
* BF2* (MHC I)	1.74	1.04E-02	1.76	3.39E-02
* B2M* (MHC I)	1.87	2.13E-03		
* CD1B* (MHC I)	2.59	3.16E-02		
Other				
* TLR1A*	3.24	3.61E-02		
* CR1*	2.60	3.79E-02		

LFC: log_2_ fold-change; FDR: false discovery rate (< 0.05 was considered significant).

### Biological pathways involved in T cell receptor signalling were enriched with up-regulated genes in the vaccinated-challenged chickens

**Figure 3:**
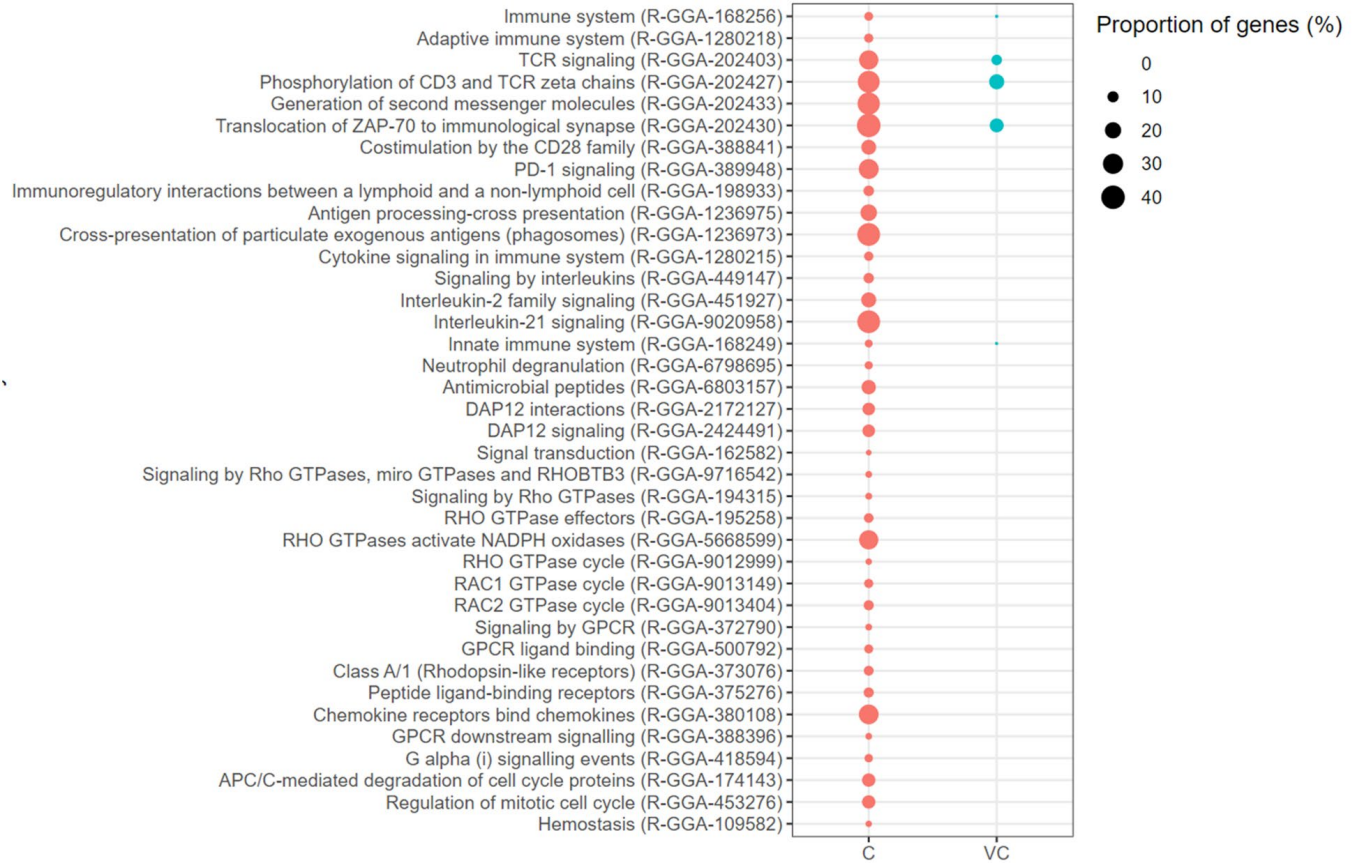
**Biological pathways enriched with up-regulated genes.** C: the unvaccinated-challenged chickens compared to the unvaccinated-unchallenged chickens. VC: the vaccinated-challenged chickens compared to the unvaccinated-unchallenged chickens. The size of the bubble indicates the proportion of the genes in the pathway that were up-regulated.

Five biological pathways were enriched with up-regulated genes in the vaccinated-challenged chickens, while 38 biological pathways were enriched with up-regulated genes in the unvaccinated-challenged chickens (Figure 3). The biological pathways enriched with up-regulated genes in the vaccinated-challenged chickens were TCR Signalling (9.09% of genes up-regulated), Phosphorylation of CD3 and TCR Zeta Chains (17.39% of genes up-regulated), Translocation of ZAP-70 to Immunological Synapse (15.0% of genes up-regulated), Innate Immune System (1.21% of genes up-regulated) and Immune System (1.34% of genes up-regulated). The first three pathways play a role in signal 1 of T cell receptor (TCR) signalling. TCR signalling-associated pathways were also enriched with up-regulated genes in the unvaccinated-challenged chickens, but with a higher proportion of genes up-regulated and with additional pathways enriched with up-regulated genes (Figure 3). The additional pathways were Generation of Second Messenger Molecules (36% of genes up-regulated), Co-stimulation by the CD28 Family (16.95% of genes up-regulated) and PD-1 Signalling (29.17% of genes up-regulated). The latter two pathways are important in signal 2 of TCR signalling.

In addition, biological pathways associated with antigen presentation, cytokine signalling, inflammation, RHO GTPase signalling, GPCR signalling and cell proliferation were enriched with up-regulated genes in the unvaccinated-challenged chickens (Figure 3). None of these biological pathways were enriched with up-regulated genes in the vaccinated-challenged chickens.

### T cell response-, B cell response- and IL4 production-associated GO terms were enriched with up-regulated genes in the vaccinated-challenged chickens

In total, 110 GO terms (103 BPs and 7 CCs) were enriched with up-regulated genes in the vaccinated-challenged chickens. In the unvaccinated-challenged chickens, 388 GO terms (357 BPs, 17 CCs and 14 MFs) were enriched with up-regulated genes. A lower proportion of genes were up-regulated in the enriched GO terms in the vaccinated-challenged chickens, where the same GO terms were enriched with up-regulated genes in both the unvaccinated-challenged and the vaccinated-challenged chickens. The broad groups of enriched GO terms in the BP category (GO-BP) included immune response, antigen recognition, chemotaxis, cytokine response, cell death and phagocytosis, leukocyte-mediated immunity, lymphocyte-mediated immunity, T-cell-mediated immunity, B-cell-mediated immunity, somatic recombination of immune receptors and signal transduction.

**Figure 4:**
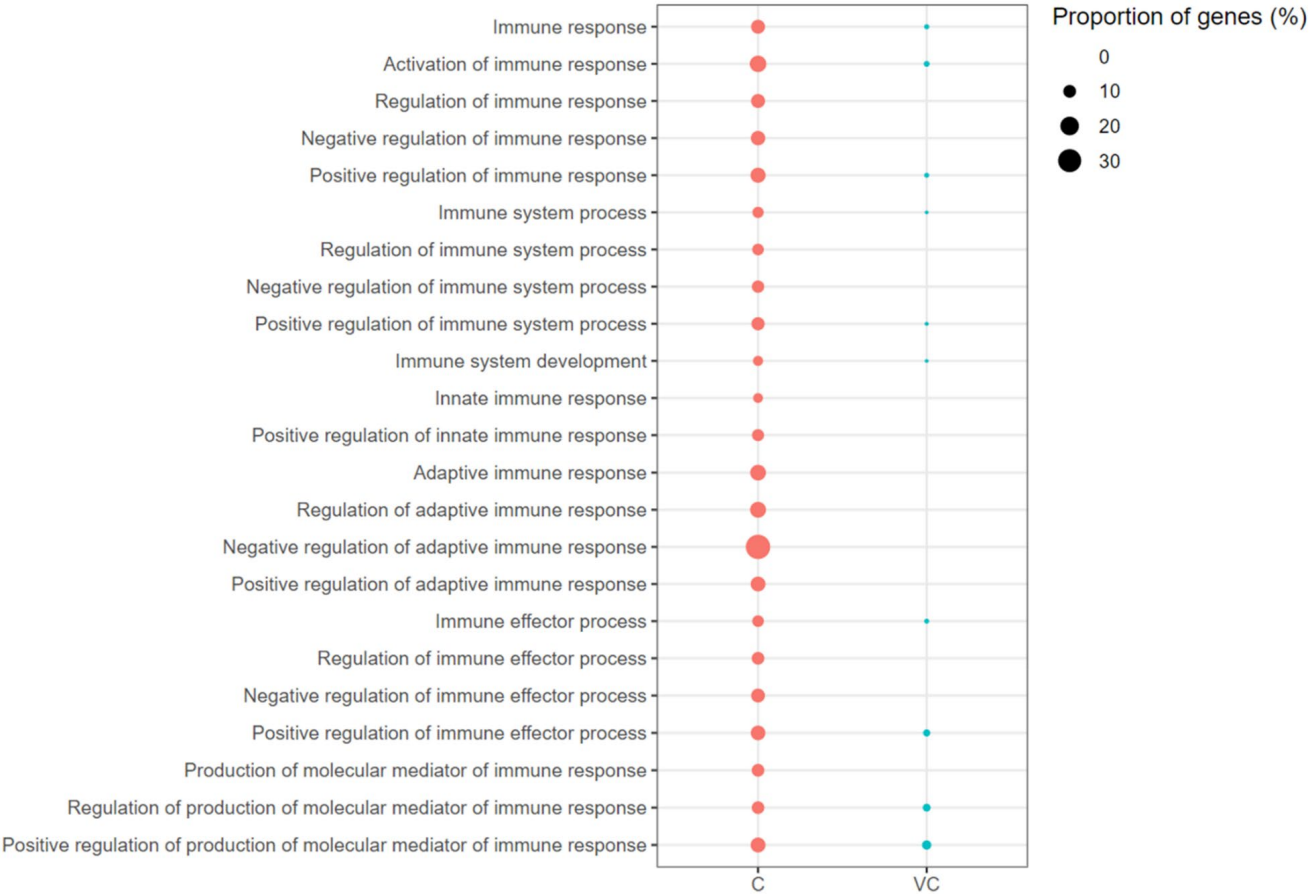
**Immune response associated gene ontology terms (biological processes) enriched with up-regulated genes.** C: the unvaccinated-challenged chickens compared to the unvaccinated-unchallenged chickens. VC: the vaccinated-challenged chickens compared to the unvaccinated-unchallenged chickens. The size of the bubble indicates the proportion of the genes in the GO term that were up-regulated.

Immune response-associated GO-BPs were enriched with up-regulated genes in both the vaccinated-challenged (10 terms) and the unvaccinated-challenged (23 terms) chickens. The GO terms Immune Response, Immune System Process, Immune System Development, and Immune Effector Process, and the terms associated with their positive regulation, were enriched in the vaccinated-challenged chickens. In addition to these, terms associated with their negative regulation, Innate Immune Response, and Adaptive Immune Response, and the terms associated with their positive and negative regulation, were enriched with up-regulated genes in the unvaccinated-challenged chickens. In the unvaccinated-challenged chickens, the enriched term with the highest proportion of up-regulated genes was Negative Regulation of the Adaptive Immune Response (Figure 4). GO terms associated with the negative regulation of the immune response were not enriched with up-regulated genes in the vaccinated-challenged chickens.

**Figure 5:**
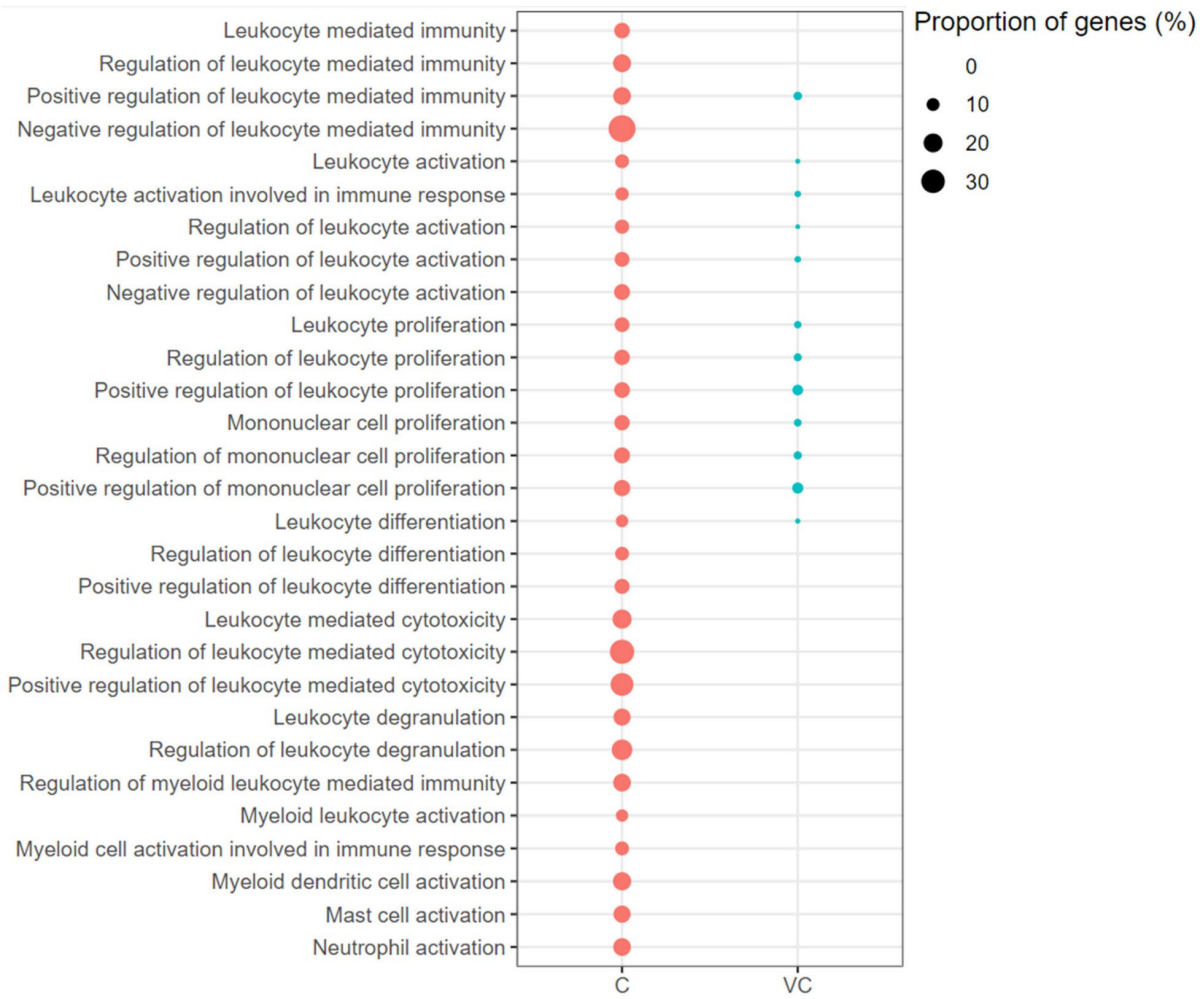
**Leukocyte mediated immunity associated gene ontology terms (biological processes) enriched with up-regulated genes.** C: the unvaccinated-challenged chickens compared to the unvaccinated-unchallenged chickens. VC: the vaccinated-challenged chickens compared to the unvaccinated-unchallenged chickens. The size of the bubble indicates the proportion of the genes in the GO term that were up-regulated.

**Figure 6:**
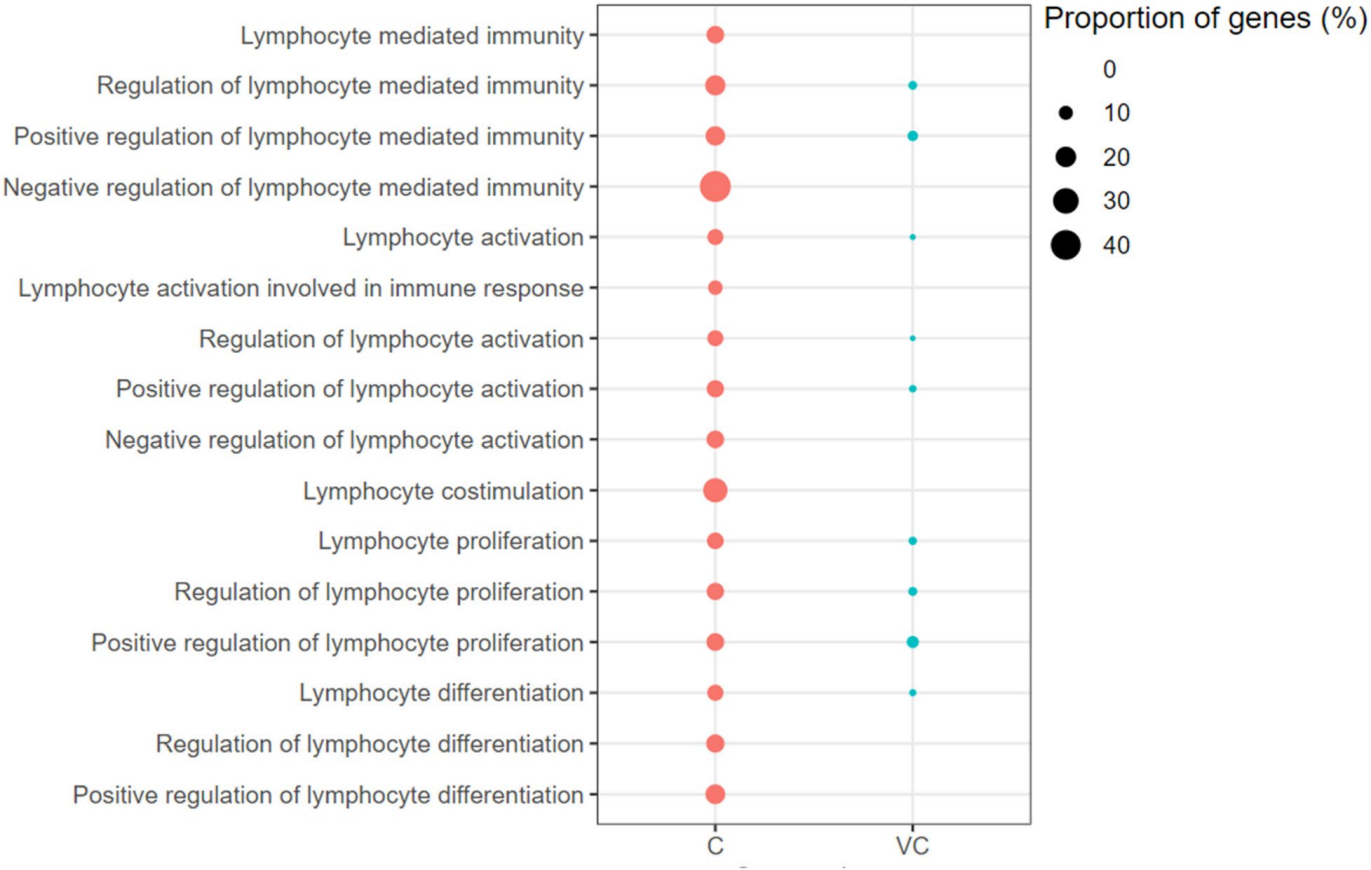
**Lymphocyte mediated immunity associated gene ontology terms (biological processes) enriched with up-regulated genes.** C: the unvaccinated-challenged chickens compared to the unvaccinated-unchallenged chickens. VC: the vaccinated-challenged chickens compared to the unvaccinated-unchallenged chickens. The size of the bubble indicates the proportion of the genes in the GO term that were up-regulated.

Leukocyte- and lymphocyte-mediated immunity-associated GO-BP terms were enriched with up-regulated genes in both the vaccinated-challenged and the unvaccinated-challenged chickens. Leukocyte Activation and its positive regulation, Leukocyte Proliferation and its positive regulation, Mononuclear Cell Proliferation and its positive regulation, and Leukocyte Differentiation were enriched with up-regulated genes in the vaccinated-challenged chickens (Figure 5). In addition to these, Negative Regulation of Leukocyte Activation, Positive Regulation of Leukocyte Differentiation, Leukocyte-mediated Cytotoxicity and its positive regulation, Leukocyte Degranulation, Myeloid Dendritic Cell Activation, Mast Cell Activation and Neutrophil Activation were enriched with up-regulated genes in the unvaccinated-challenged chickens (Figure 5). The enriched GO term with the highest proportion of up-regulated genes in the unvaccinated-challenged chickens was Negative Regulation of Lymphocyte-mediated Immunity (42.86%). In contrast, negative regulatory terms were not enriched with up-regulated genes in the vaccinated-challenged chickens. Similarly, Lymphocyte Co-stimulation was enriched with up-regulated genes only in the unvaccinated-challenged birds, and not in the vaccinated-challenged chickens (Figure 6).

**Figure 7:**
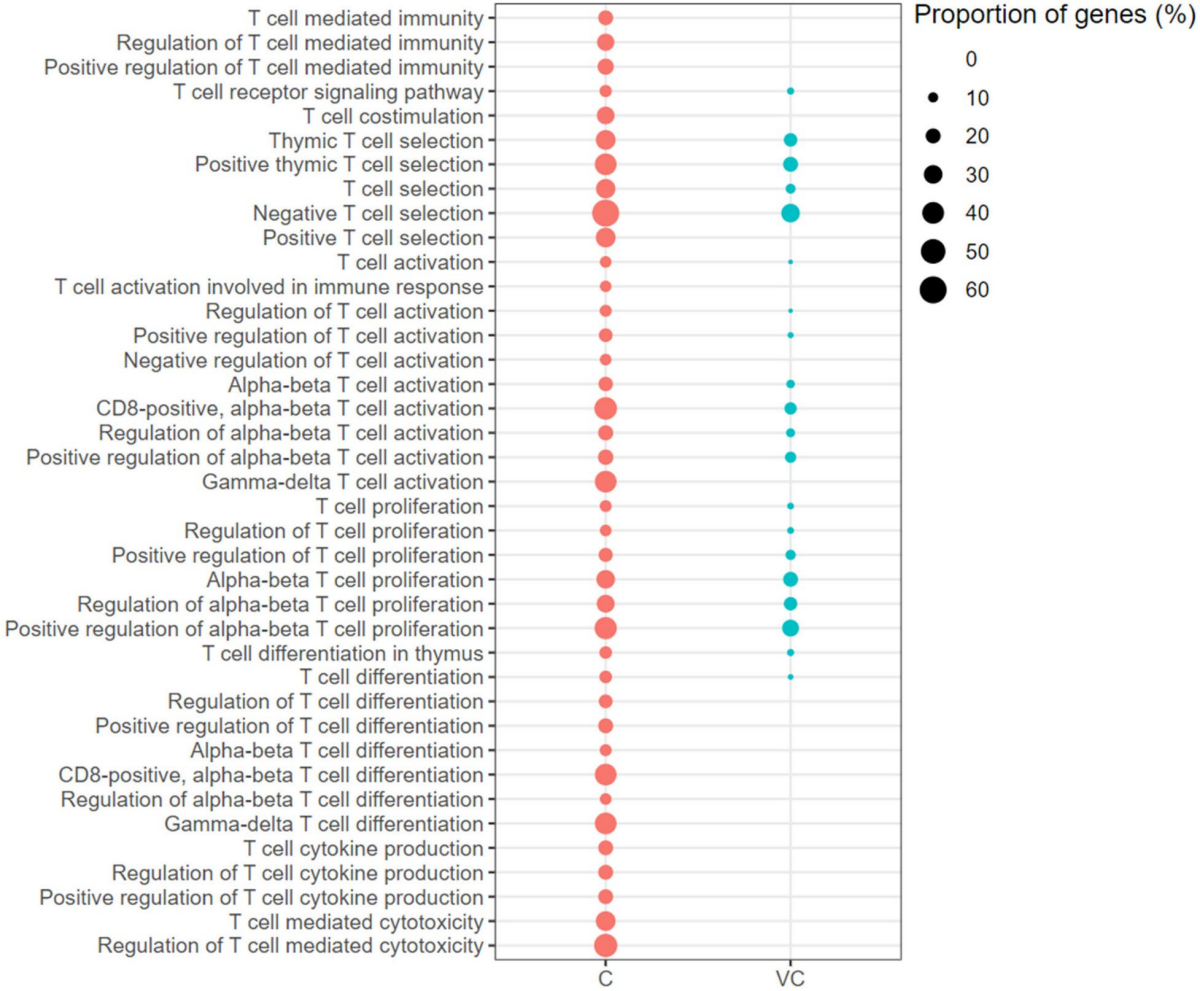
**T-cell mediated immunity associated gene ontology terms (biological processes) enriched with up-regulated genes.** C: the unvaccinated-challenged chickens compared to the unvaccinated-unchallenged chickens. VC: the vaccinated-challenged chickens compared to the unvaccinated-unchallenged chickens. The size of the bubble indicates the proportion of the genes in the GO term that were up-regulated.

Twenty GO-BP terms associated with TCR Signalling, T-cell Selection, T-cell Activation, T-cell Proliferation and T-cell Differentiation were enriched with up-regulated genes in the vaccinated-challenged chickens (Figure 7). Furthermore, terms associated with the Positive Regulation of T cell Activation and Proliferation were also enriched. The GO terms that were not enriched with up-regulated genes in the vaccinated-challenged chickens, but that were enriched with them in the unvaccinated-challenged chickens included T-cell Co-stimulation, Negative Regulation of T-cell Activation, Positive Regulation of T-cell Differentiation, T-cell Cytokine Production, Positive Regulation of T-cell Cytokine Production and T-cell Mediated Cytotoxicity (Figure 7).

**Figure 8:**
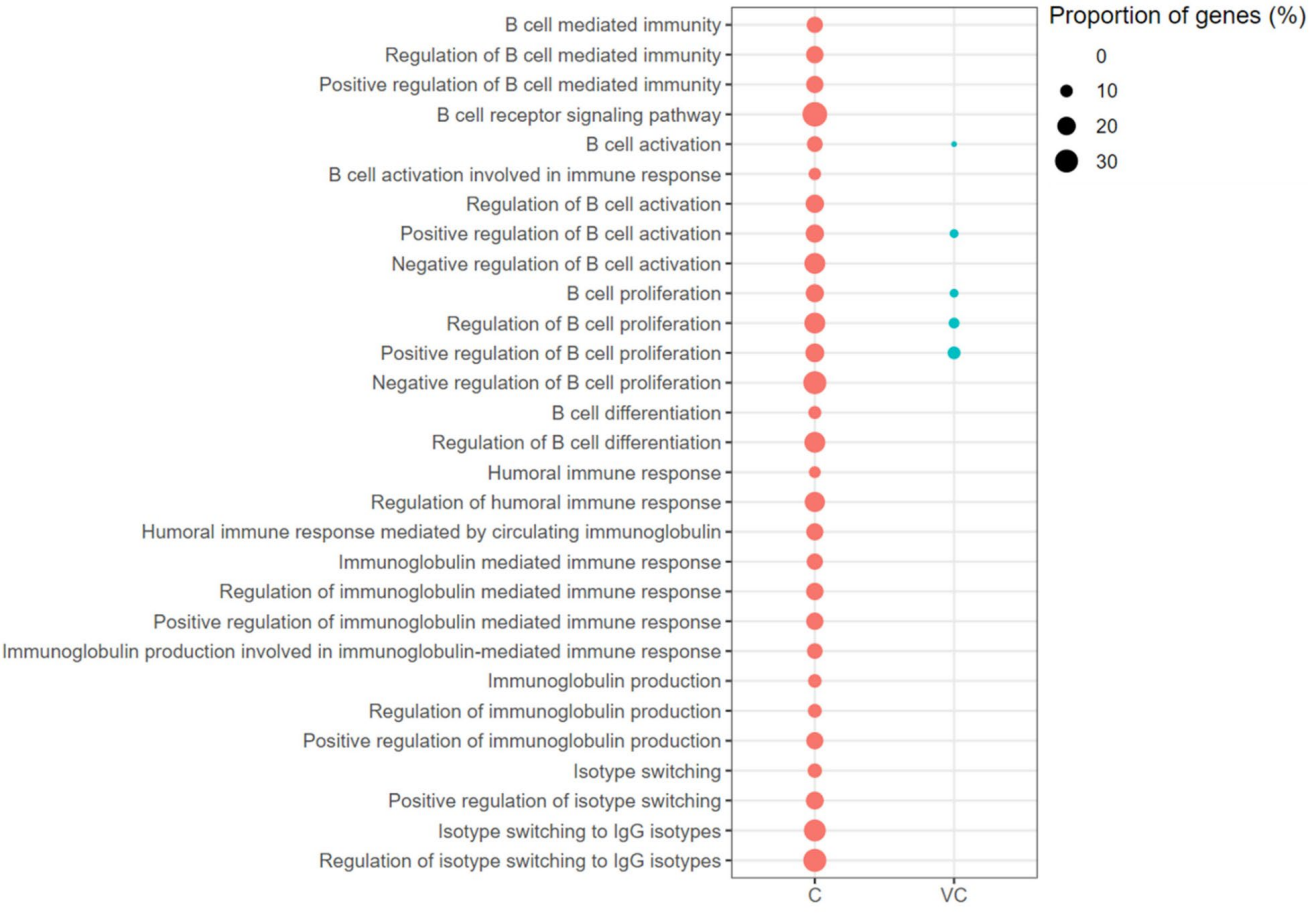
**B-cell mediated immunity associated gene ontology terms (biological processes) enriched with up-regulated genes.** C: the unvaccinated-challenged chickens compared to the unvaccinated-unchallenged chickens. VC: the vaccinated-challenged chickens compared to the unvaccinated-unchallenged chickens. The size of the bubble indicates the proportion of the genes in the GO term that were up-regulated.

B-cell Activation (2.60% genes), Positive Regulation of B-cell Activation (5.36% genes), B-cell Proliferation (5.26% genes) and Positive Regulation of B-cell Proliferation (10.34% genes) were the GO-BP terms enriched with up-regulated genes in the vaccinated-challenged chickens. These terms were also enriched with up-regulated genes in the unvaccinated-challenged chickens, but a higher proportion of genes associated with each of terms were up-regulated (Figure 8). Apart from these, Negative Regulation of B-cell Activation, Negative Regulation of B-cell Proliferation (with a higher proportion of genes involved in negative regulation up-regulated than the proportion of genes involved in positive regulation that were up-regulated) and B-cell Differentiation were enriched with up-regulated genes in the unvaccinated-challenged chickens. Importantly, isotype switching-associated GO terms were enriched with up-regulated genes only in the unvaccinated-challenged chickens, and not in the vaccinated-challenged chickens (Figure 8).

**Figure 9:**
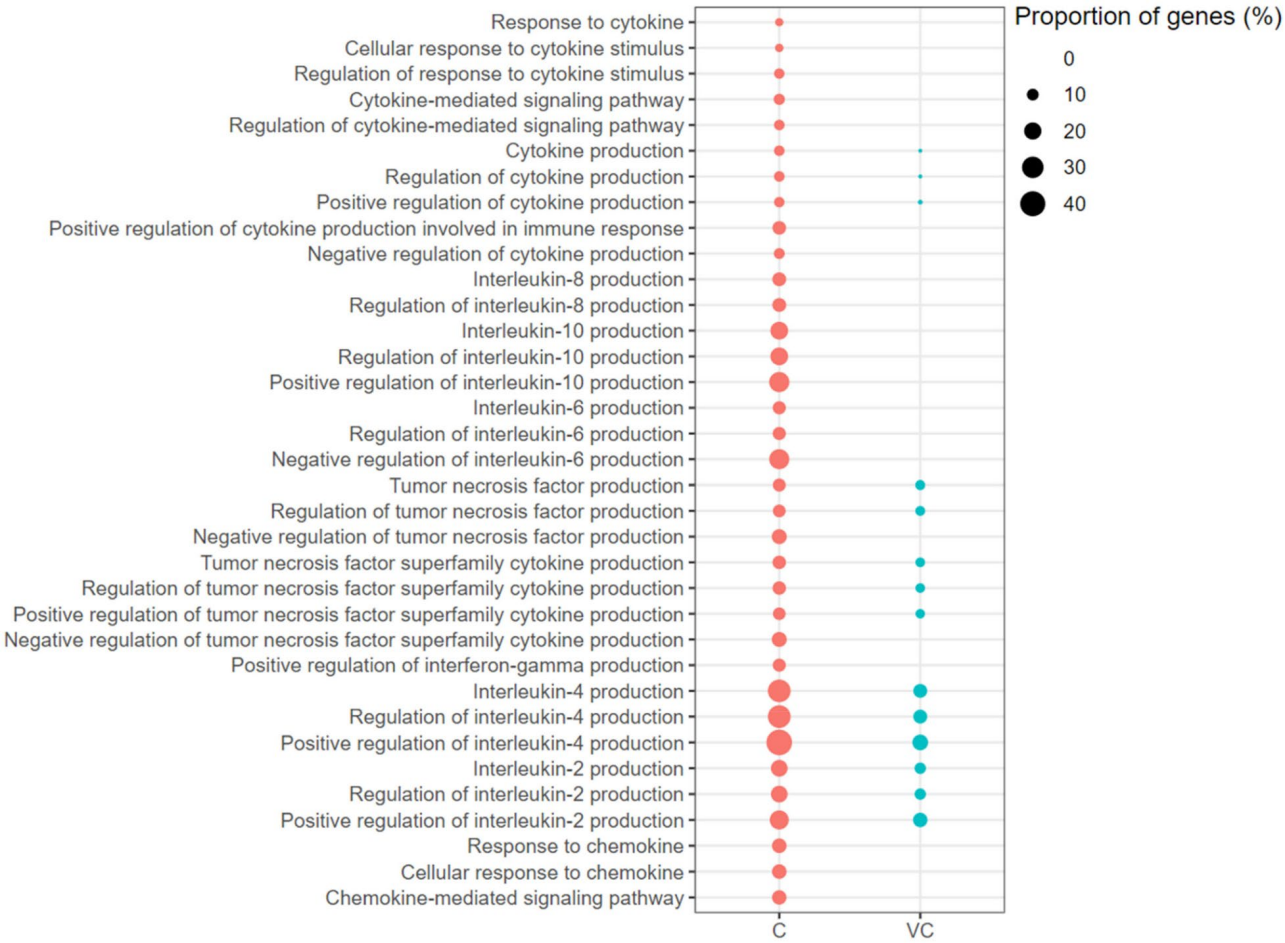
**Cytokine response associated gene ontology terms (biological processes) enriched with up-regulated genes.** C: the unvaccinated-challenged chickens compared to the unvaccinated-unchallenged chickens. VC: the vaccinated-challenged chickens compared to the unvaccinated-unchallenged chickens. The size of the bubble indicates the proportion of the genes in the GO term that were up-regulated.

The GO-BP terms associated with IL4 Production, IL2 Production, and Tumour Necrosis Factor Production, and their positive regulation, were enriched with up-regulated genes in both the vaccinated-challenged and the unvaccinated-challenged chickens (Figure 9). The GO-BP terms associated with IL8 Production, IL10 Production and its positive regulation, IL6 Production and its negative regulation, and Positive Regulation of Interferon-gamma Production were enriched with up-regulated genes only in the unvaccinated-challenged birds, and not in the vaccinated-challenged chickens (Figure 9).

**Figure 10:**
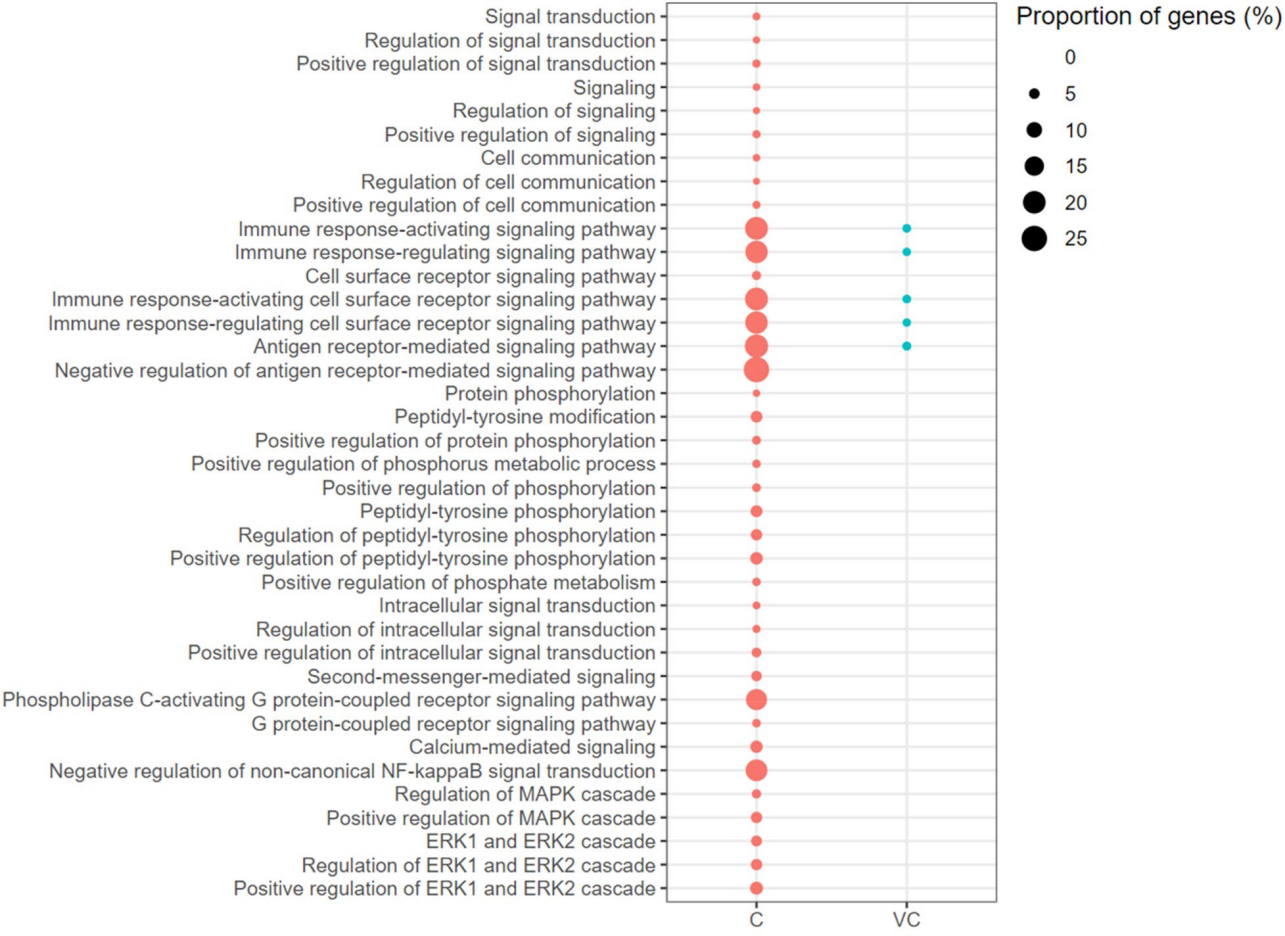
**Signal transduction associated gene ontology terms (biological processes) enriched with up-regulated genes. **C: the unvaccinated-challenged chickens compared to the unvaccinated-unchallenged chickens. VC: the vaccinated-challenged chickens compared to the unvaccinated-unchallenged chickens. The size of the bubble indicates the proportion of the genes in the GO term that were up-regulated.

Among the signal transduction-associated GO-BP terms, five terms related to cell surface receptor signalling were enriched with up-regulated genes in the vaccinated-challenged chickens (Figure 10). The terms Negative Regulation of Non-canonical NF-κB Signal Transduction (18.75% of genes up-regulated), Positive Regulation of the MAPK Cascade (5.71% genes of genes up-regulated), and Positive Regulation of the ERK1 and ERK2 Cascade (7.21% of genes up-regulated) were enriched with up-regulated genes only in the unvaccinated-challenged chickens (Figure 10).

GO-BP terms associated with antigen recognition (8 terms), chemotaxis (29 terms), somatic recombination of immune receptors (10 terms), cell death and phagocytosis (25 terms) were enriched with up-regulated genes in the unvaccinated-challenged chickens (Additional file 1). In the chemotaxis group, the term with highest proportion of up-regulated genes was Positive Regulation of Neutrophil Migration (28.57% of genes up-regulated) (Additional file 1). In the somatic recombination of immune receptors group, terms associated with both the Positive and the Negative regulation of Somatic Recombination of Immune Receptors were enriched with up-regulated genes, with a higher proportion of up-regulated genes in the term Negative Regulation of Somatic Recombination of Immune Receptors (Additional file 1). None of these GO-BP terms were enriched with up-regulated genes in the vaccinated-challenged chickens.

Fourteen GO terms in the category of MFs were enriched with up-regulated genes only in the unvaccinated-challenged chickens. These terms were mainly associated with cytokine and chemokine receptor activity and binding. In addition, Oxidoreductase Activity, Acting on NAD(P)H, Oxygen as Acceptor, Non-membrane Spanning Protein Tyrosine Kinase Activity and MHC Protein Binding were also enriched with up-regulated genes (Additional file 2).

The GO terms enriched with up-regulated genes in the category of CC in the vaccinated-challenged chickens were mainly associated with the T cell receptor complex. In addition, the terms MHC Protein Complex, Immunological Synapse, Immunoglobulin Complex and Podosome were enriched with up-regulated genes in the unvaccinated-challenged chickens (Additional file 3).

### No significant functional enrichment with down-regulated genes in the vaccinated-challenged chickens

The down-regulated genes in the vaccinated-challenged chickens are listed in Additional file 4. There were no biological pathways, GO-BP terms or GO-MF terms enriched with these down-regulated genes. The GO-CC term, Extracellular Region, was enriched with down-regulated genes (FDR = 0.02), but the proportion of genes involved was less than 1%. The biological pathway Regulation of TLR by Endogenous Ligand was enriched with down-regulated genes (FDR = 8.87E-03, proportion of genes down-regulated = 19%) in the unvaccinated-challenged chickens. In addition, the GO-CC terms Extracellular Region (FDR = 0.03, proportion of genes down-regulated = 1.11%) and Extracellular Space (FDR = 0.04, proportion of genes down-regulated = 1.38%) were enriched with down-regulated genes in the unvaccinated-challenged chickens.

## Discussion

This study differentiated the mechanisms underlying the pathological effects of challenge with the virulent MS field strain 94011 V-18d in unvaccinated and Vaxsafe MS-vaccinated chickens. The assessment and comparison of TMTs of the three experimental groups showed that vaccination protected against tracheal mucosal pathology. The transcriptional changes in the tracheal mucosa of the vaccinated-challenged chickens were consistent with an efficient secondary immune response. Furthermore, pathology-related transcriptional changes were absent in the vaccinated-challenged chickens.

Compared to the unvaccinated-unchallenged negative control chickens, the down-regulation of the *NR4A3* gene in the vaccinated-challenged chickens, but not in the unvaccinated-challenged chickens, may be important in vaccine-mediated protection. This gene is a transcriptional activator that can impede the B-cell response to antigens following its induction through B-cell receptor stimulation [29]. In addition, up-regulation of *NR4A3* has been shown to limit the generation of central memory CD8 T-cells, while enhancing short-lived effector CD8 T-cell differentiation [30]. Hence, its down-regulation in the vaccinated-challenged chickens may indicate an efficient B cell-response and/or enhanced memory CD8 T-cell generation. The *PDK4* gene is associated with inflammation [31–33]. Therefore, its unique down-regulation in the vaccinated-challenged chickens compared to the unvaccinated-unchallenged negative control chickens may indicate its involvement in the prevention of inflammation in the vaccinated birds following challenge. Although the exact role of the down-regulation of the *WNT9A* gene in the vaccinated-challenged birds is not clear, members of the Wnt protein family are also involved in the regulation of inflammation and are considered promising therapeutic targets to reduce inflammation [34]. The uniquely up-regulated gene in the vaccinated-challenged birds that encodes an uncharacterised protein may also have a role in vaccine-mediated protection against *M. synoviae* infection. The objective of the studies described here was to provide an overview of global tracheal mucosal responses to *M. synoviae* infection in vaccinated chickens. The genes identified by these studies that may play a role in protection need to be further assessed, preferably in a time course experiment specifically examining their expression by RT-qPCR throughout the course of infection, before drawing definitive conclusions. Future studies will also need to characterise the function of these genes further to determine if they have specific roles in inducing vaccine-mediated protection.

Differentially transcribed genes and enriched functional categories reflecting persistent and/or higher antigenic stimuli were evident only in the unvaccinated-challenged chickens. These included 6 GO terms indicative of a response to bacterial cell membrane components, 2 GO terms indicative of enhanced toll-like receptor signalling, up-regulated genes encoding MHC I and II class molecules, MHC-associated GO terms, and biological pathways associated with antigen processing-cross presentation. Among these, only the GO term Response to Bacterium and the gene encoding the MHC class I alpha 2 domain were enriched in the vaccinated-challenged chickens. These differences were indicative of a lower pathogen load in the vaccinated chickens than in the unvaccinated chickens two weeks after challenge. In a previous study that evaluated the protection provided by the GT5 vaccine against infection with the virulent *Mycoplasma gallisepticum* strain R_low_, early induction of *M. gallisepticum*-specific IgG and IgA immunoglobulins was evident in vaccinated birds [35]. It has been proposed that this early appearance of specific immunoglobulins and their local mucosal secretion and/or transudation onto mucosal surfaces could reduce tracheal colonisation by *M. gallisepticum* and result in more clear rapid clearance of the organism [35, 36]. A similar mechanism may have contributed to the protection induced by the Vaxsafe MS vaccine, reducing the replication and persistence of the challenge strain and thereby preventing most of the above transcriptional changes observed in the unvaccinated-challenged birds.

The most prominent transcriptional changes in the vaccinated-challenged chickens included the genes and the functional categories associated with T-cell responses. These transcriptional changes were also present in the unvaccinated-challenged chickens, but with important differences. While T-cell receptor signalling was enriched with up-regulated genes in both the vaccinated-challenged and the unvaccinated-challenged chickens, T-cell co-stimulation was enriched with up-regulated genes only in the unvaccinated-challenged chickens. It is widely accepted that co-stimulation is necessary for the activation of naïve CD4 and CD8 T cells during a primary T cell response. However, the role of co-stimulation during a secondary T-cell response is controversial [37]. While some studies have suggested that co-stimulation is not required for memory CD4 and CD8 T-cell activation [38–41], others have found that co-stimulation has a critical role in their optimal activation [42–44]. Given the lower activation threshold of memory T-cells, due to their higher antigenic affinity [45, 46], co-stimulation may not be necessary for their complete activation. Moreover, functional heterogeneity of memory T-cells is believed to be a contributor to discrepancies seen in the literature [47]. In the study reported here, the transcriptional changes indicative of T-cell co-stimulation seen in the unvaccinated-challenged chickens correlated with a primary T-cell response, while the absence of these transcriptional changes in the vaccinated-challenged chickens is probably indicative that co-stimulation was not required for the memory response 2 weeks after infection.

There were some differences in proximal TCR signalling between the vaccinated-challenged and the unvaccinated-challenged chickens, which was also indicative of respective memory and primary T-cell responses in the two groups. Although the biological pathway Phosphorylation of CD3 and TCR Zeta Chains was enriched with up-regulated genes in both groups, the gene *ZAP70* was up-regulated only in the unvaccinated-challenged group. It has been shown that, unlike naïve CD4 T-cells, memory CD4 T-cells do not phosphorylate ZAP-70, but instead phosphorylate a ZAP-70-related kinase, p72syk [48]. Furthermore, increased expression of the adapter molecule SLP-76 has been seen in naïve and effector CD4 T-cells, while reduced levels of SLP-76 were seen in the memory CD4 T cells [49]. In the studies described here, the *SLP76* gene was up-regulated only in the unvaccinated-challenged chickens, and not in the vaccinated-challenged chickens. These results further confirmed a primary response involving naïve T-cells in the unvaccinated-challenged chickens compared to a secondary response involving memory T-cells in the vaccinated-challenged chickens.

The up-regulation of genes *IL2RG* and *IL21R*, and the enrichment of the GO terms IL4 Production and Positive Regulation of IL4 Production with up-regulated genes, indicate the involvement of T follicular helper (T_FH_) cells in the vaccinated-challenged chickens. The two major sources of IL4 are T helper 2 (T_H_2) and T follicular helper (T_FH_) cells [50–52]. However, *GATA3* and *STAT6* expression is required for IL4 production from T_H_2 cells [50, 52] and these were not differentially transcribed in the vaccinated-challenged chickens. Moreover, IL21 is regarded as a signature cytokine secreted by T_FH_ cells, binding to heterodimers of IL21R and IL2RG receptors on these cells [53, 54]. T_FH_ cells play an important role in B-cell differentiation and germinal centre formation in both primary and secondary humoral responses [55]. The up-regulation of the *TNFRSF13C* gene encoding the BAFFR receptor and the enrichment with up-regulated genes of GO terms associated with TNF/TNFSF cytokine production in the vaccinated-challenged chickens further indicated a humoral response, as these cytokines and their receptors play a role in B-cell development and survival [56, 57]. In addition, the gene encoding IL16 was also up-regulated in the vaccinated-challenged chickens. Follicular B lymphocytes are known to express IL16 to recruit T helper cells [58]. Therefore, up-regulation of the *IL16* gene in the vaccinated-challenged chickens may also be associated with the humoral immune response. Although the current study did not aim to characterise immune cells infiltrating the tracheal mucosa, a recent study identified CD4^+^CD25^−^ and Bu1^+^ cells in the tracheal mucosa of both Vaxsafe MS vaccinated and unvaccinated chickens following challenge with *M. synoviae* strain 94011/v-18d, starting from 2 days after challenge [12]. Taken together, it is clear that a T-cell dependent B-cell response is present in both vaccinated and unvaccinated birds 14 days after challenge with *M. synoviae* strain 94011/v-18d.

While the B cell response was evident in both vaccinated and unvaccinated birds, the analysis of the GO terms revealed an antigen-specific secondary B-cell response in vaccinated birds and a primary B-cell response in the unvaccinated birds. This was demonstrated by the lack of enrichment with up-regulated genes of the GO terms associated with immunoglobulin isotype switching and somatic diversification of immune receptors in the vaccinated-challenged chickens, as the B-cells involved in a secondary humoral immune response are already class-switched and affinity-matured [55].

In the current study, the GO terms CD8-positive, Alpha–beta T-cell Activation, and CD8-positive, Alpha–beta T-cell Differentiation were enriched with up-regulated genes in the vaccinated-challenged chickens, also indicating a role of CD8 cells at 14 days after challenge. A recent study detected CD4^−^CD8^+^ cells in the tracheal mucosa of chickens vaccinated with Vaxsafe MS at 7 and 21 days after challenge with *M. synoviae* strain 94011/v-18d [12]. However, these birds also showed enhanced transcription of *IFNG* in the tracheal mucosa at 7 and 21 days after challenge [12], whereas *IFNG* was not differentially transcribed in vaccinated-challenged chickens in the study described here, and the functional categories associated with it were not enriched with up-regulated genes in the vaccinated-challenged chickens. These observations may indicate that a B-cell mediated humoral response is prominent at 14 days after challenge, in contrast to a CD8+ T-cell mediated cytotoxic effect seen at 7 and 21 days after challenge. It is also worthwhile mentioning that these differences may have resulted from the differing experimental methods used in these two studies. In particular, the current study used a *M. synoviae* strain 94011/v-18d and IBV field strain V1/71 co-challenge model, whereas the previous study used only *M. synoviae* strain 94011/v-18d as the challenge. Moreover, the current study employed mRNA sequencing and global transcriptional profiling, in contrast to the targeted qPCR assays used in the earlier study [12], and these two techniques can differ in sensitivity and specificity [59].

A distinct set of transcriptional changes indicative of T helper 1 (T_H_1) polarisation was evident in the unvaccinated-challenged chickens, but not in the vaccinated-challenged chickens. Upon activation of naïve CD4 T cells, IL12 activates STAT4, which then initiates a T_H_1 response by enhancing IFN gamma production [60, 61]. IFN gamma production by T_H_1 cells has also been linked with the activation of MAPK cascades [62]. Hence, the up-regulation of the *IL12RB1* and *STAT4* genes and the enrichment with up-regulated genes of the GO terms Positive Regulation of IFNG Production and Positive Regulation of MAPK/ERK Cascades in the unvaccinated-challenged chickens was indicative of a T_H_1 response leading to inflammation. The enrichment with up-regulated genes of the GO term Negative Regulation of IL6 Production also indicated a T_H_1 response in the unvaccinated-challenged chickens. IL6 is an important regulator of T_H_1/T_H_2 differentiation, where it inhibits the T_H_1 differentiation [63]. Therefore, its negative regulation is likely to facilitate T_H_1 differentiation.

Inflammation-associated transcriptional changes, such as enrichment of neutrophil activation, neutrophil degranulation, DAP12 interactions, DAP12 signalling, chemotaxis, phagocytosis, cell killing and *IL8* up-regulation, were detected in the unvaccinated-challenged chickens, but not in the vaccinated-challenged chickens. Furthermore, toll-like receptor signalling was enriched with up-regulated genes only in the unvaccinated-challenged birds, and not in the vaccinated-challenged chickens. Toll-like receptor signalling is crucial in the establishment of inflammation following mycoplasma infections [64, 65]. These results demonstrate protection by the Vaxsafe MS vaccine against T_H_1 polarisation and the establishment of chronic inflammation, most likely through a rapid, antibody-mediated reduction of the pathogen load, restricting stimulation of toll-like receptors.

A further difference between the vaccinated-challenged and unvaccinated-challenged chickens was the lack of immune dysregulation-associated transcriptional changes in the vaccinated birds. Increased levels of IL10 have been associated with mycoplasma infections and this is believed to have a role in the pathogenesis of mycoplasmosis [66–68], most likely by inducing immunosuppression. In agreement with this, transcription of the *IL10RA* gene was up-regulated and the GO terms IL10 Production and Positive Regulation of IL10 Production were enriched with up-regulated genes only in the unvaccinated-challenged chickens. Chronic infections have been associated with defective T-cell responses mediated by PD-1 signalling [69]. PD-1 signalling-associated transcriptional changes were evident only in the unvaccinated-challenged chickens, highlighting the role of a PD-1 signalling mediated dysregulated T-cell response in the pathogenesis of disease caused by *M. synoviae*. The *FCRL4* gene, which inhibits B-cell receptor signalling, was up-regulated in the unvaccinated-challenged chickens. Prolonged exposure to high loads of antigen can result in B-cell exhaustion through FCRL4-mediated inhibitory signals [55]. Furthermore, the enrichment with up-regulated genes of the GO term Negative Regulation of Non-canonical NF-κB Signal Transduction in the unvaccinated-challenged chickens was indicative of a defective B-cell response, as non-canonical NF-κB signal transduction plays a vital role in BAFFR-mediated survival of B-cells and germinal centre formation [70]. Moreover, GO terms associated with both the positive and the negative regulation of adaptive immune response (with a higher proportion of up-regulated genes in GO terms associated with negative regulation) were enriched with up-regulated genes in the unvaccinated-challenged chickens, indicating immune dysregulation.

Although a secondary B cell response that appeared to be protective predominated in vaccinated-challenged chickens at 14 days after challenge, exploration of tracheal mucosal transcriptional changes at earlier time points after challenge will be required to confirm whether it plays a specific role in protection. It is likely that an efficient secondary humoral response promptly cleared and reduced the pathogen load in vaccinated birds, which may have led to more rapid resolution of inflammation during the acute stage of disease. Thus, the transcriptomic differences observed at 14 days after challenge may be influenced by either the absence or a reduced number of inflammatory cells in the tracheal mucosae of the vaccinated-challenged chickens. Investigating earlier time points would enable exploration of the differences between vaccinated and unvaccinated chickens after challenge in the dynamics of their inflammatory reactions. The study described here focused on the differential responses to infection with MS in the tracheal mucosae of chickens. It would be interesting to investigate transcriptional changes in response to infection with MS in other tissues in the respiratory tract, such as the nasal cavity and the air sacs, in future studies. The experiments described here did not include a vaccinated-unchallenged control group. Therefore, it was not possible to differentiate any transcriptional changes induced directly by the vaccine strain. In a previous study, the MS-H vaccine strain induced a T_H_17 response in the tracheal mucosa for up to 21 days after vaccination [11]. In the studies described here, the tracheal response was evaluated at six weeks after vaccination and no transcriptional changes indicative of a residual T_H_17 response were detected. While it is likely that the tracheal mucosal changes induced directly by the vaccine strain had completely resolved by six weeks after vaccination, we cannot completely exclude this possibility. Moreover, the mutations in the MS-H vaccine strain in the genes encoding ObgE, OppF and GAPDH are thought to, respectively, reduce horizontal transmission, reduce colonisation in the upper trachea and reduce pathogenicity in the upper trachea [71]. Reversion of these mutations to the parental strain genotype has been seen in some vaccine strain re-isolates [72]. Therefore, future studies could be conducted in chickens inoculated with the MS-H strain and with re-isolates containing original *obgE*, *oppF* or *gapdh* alleles to assess the impact of these mutations on the tracheal mucosal transcriptional profile. We have evaluated the *M. synoviae* and IBV co-infection challenge model used in the studies described here previously and have shown that infection with just *M. synoviae* or IBV does not result in major transcriptional changes in the tracheal mucosae of unvaccinated chickens at 2 weeks after infection [5]. Therefore, we did not include these groups in this experiment.

In conclusion, analysis of the tracheal mucosal transcriptional profiles of Vaxsafe MS vaccinated chickens revealed a T_FH_ cell dependent memory B-cell response two weeks after challenge with virulent MS. Moreover, the vaccine appeared to prevent or to promote rapid resolution of T_H_1-mediated inflammation and immune dysregulation-associated transcriptional changes in the tracheal mucosa. The potential role of the genes *NR4A3, PDK4* and *WNT9A* in the protection mediated by vaccination with Vaxsafe MS should be further investigated.

## Supplementary Information

**Additional file 1. Other gene ontology terms in the category of biological processes enriched with up-regulated genes. **Antigen recognition, chemotaxis, cell death and phagocytosis, somatic recombination of immune receptors associated gene ontology terms in the category of biological processes enriched with up-regulated genes in the unvaccinated-challenged and vaccinated-challenged chickens, compared to the unvaccinated-unchallenged chickens.

**Additional file 2.** **Gene ontology terms in the category of molecular functions enriched with up-regulated genes. **The gene ontology terms in the category of molecular functions enriched with up-regulated genes in the unvaccinated-challenged and vaccinated-challenged chickens, compared to the unvaccinated-unchallenged chickens.

**Additional file 3.** **Gene ontology terms in the category of cellular components enriched with up-regulated genes. **The gene ontology terms in the category of cellular components enriched with up-regulated genes in the unvaccinated-challenged and vaccinated-challenged chickens, compared to the unvaccinated-unchallenged chickens.

**Additional file 4.** **Down-regulated genes in the vaccinated-challenged chickens. **Down-regulated genes in the vaccinated-challenged chickens compared to the unvaccinated-unchallenged chickens.

## Data Availability

The datasets generated and/or analysed during the current study are available in the NCBI's Sequence Read Archive (SRA) repository under the BioProject ID PRJNA1200969.
