# Supplementary figures and images for: Mechanisms of vaccine protection in chickens against challenge with virulent Mycoplasma synoviae
Source: Vet Res. 2025 Jul 9;56:146. doi: 10.1186/s13567-025-01571-3 (PMC12243254; doi:10.1186/s13567-025-01571-3)

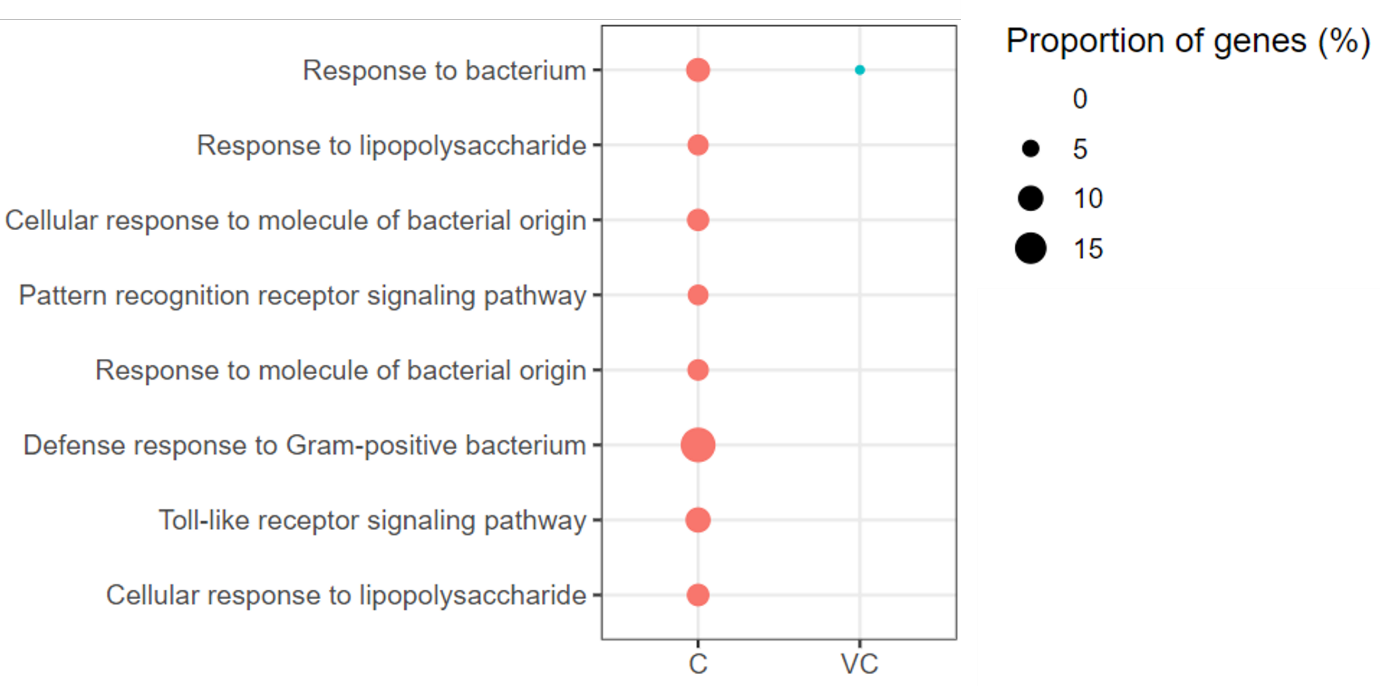


**A.**


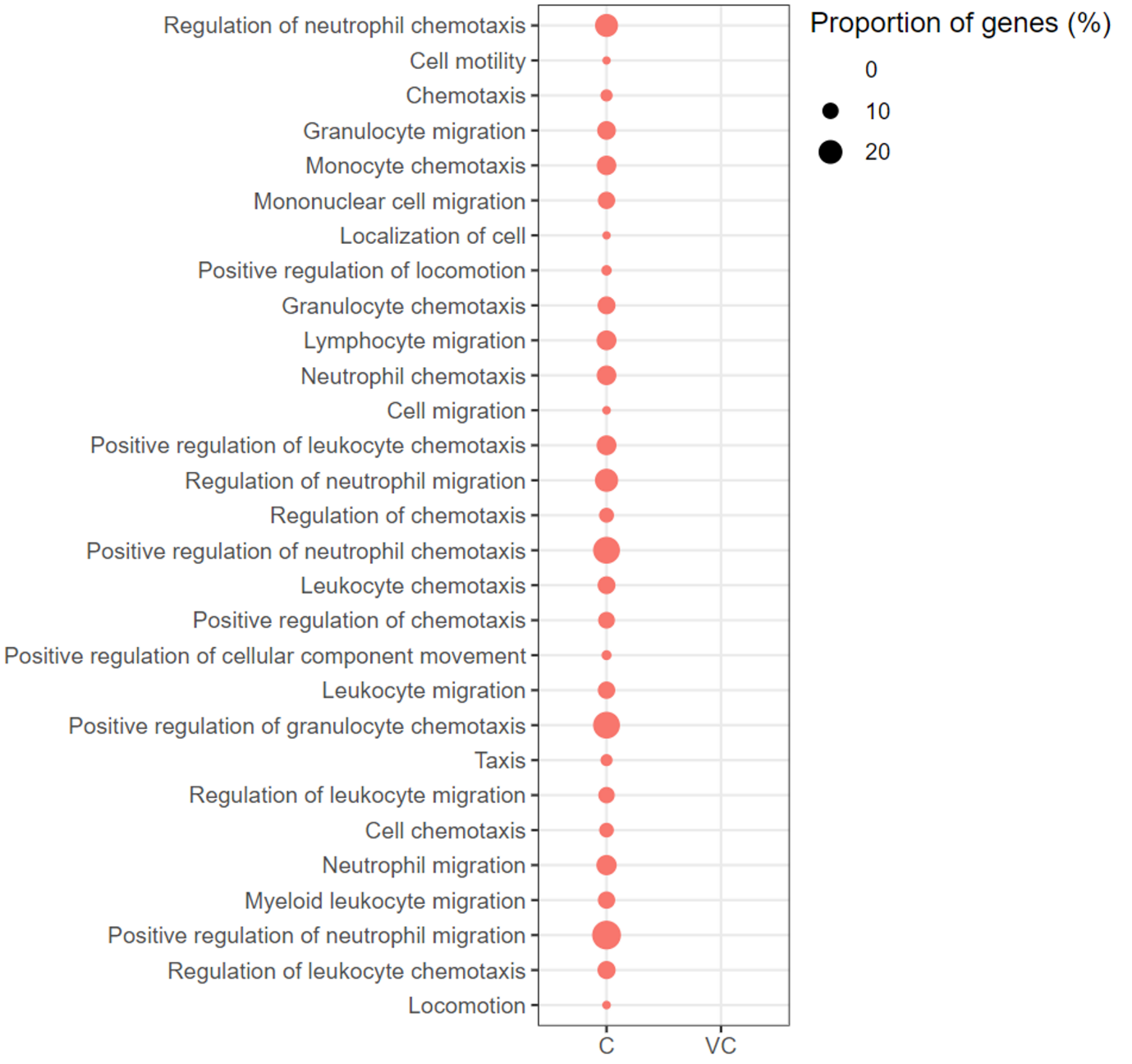


**B.**


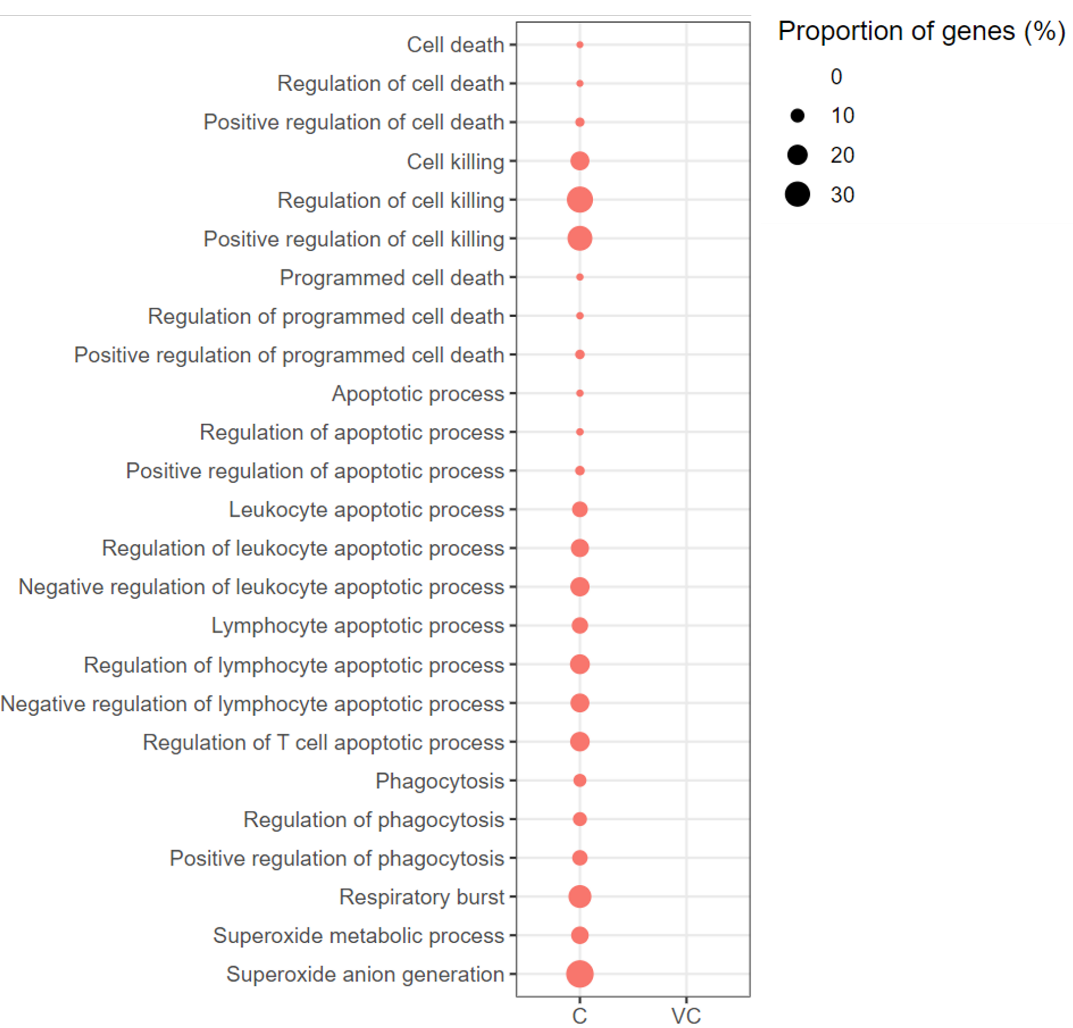


**C.**


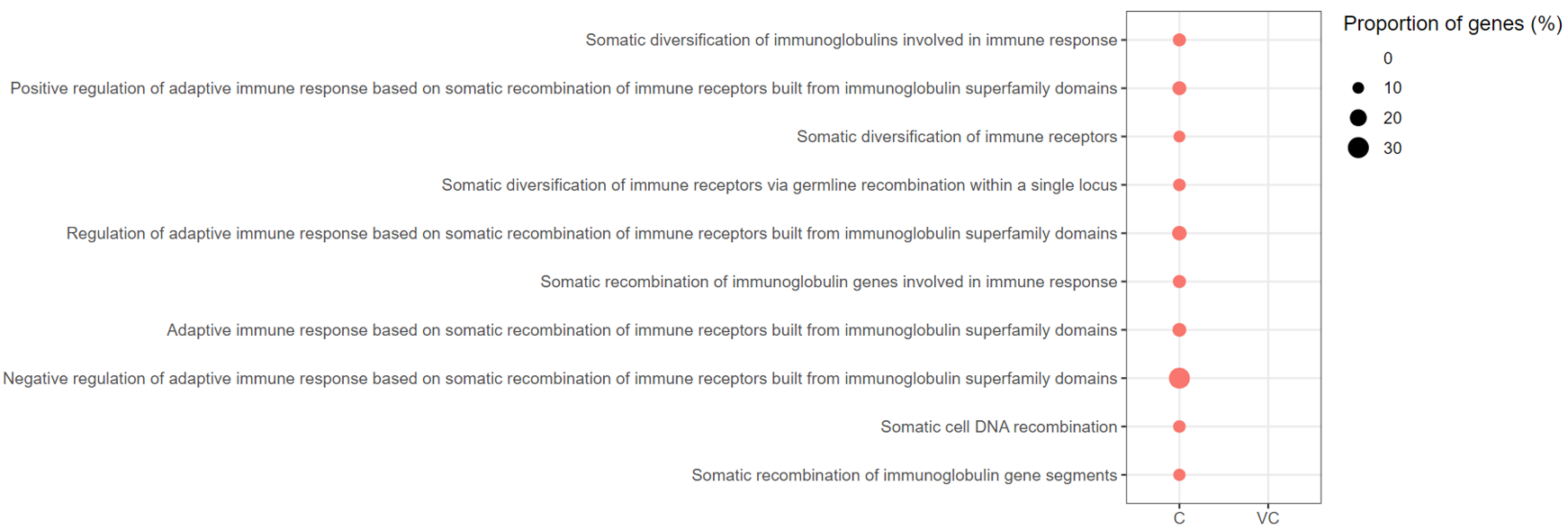


**D.**

Supplement: Supplementary file 1 — Additional file 1. Other gene ontology terms in the category of biological processes enriched with up-regulated genes. Antigen recognition, chemotaxis, cell death and phagocytosis, somatic recombination of immune receptors associated gene ontology terms in the category of biological processes enriched with up-regulated genes in the unvaccinated-challenged and vaccinated-challenged chickens, compared to the unvaccinated-unchallenged chickens. [file 13567_2025_1571_MOESM1_ESM.docx]

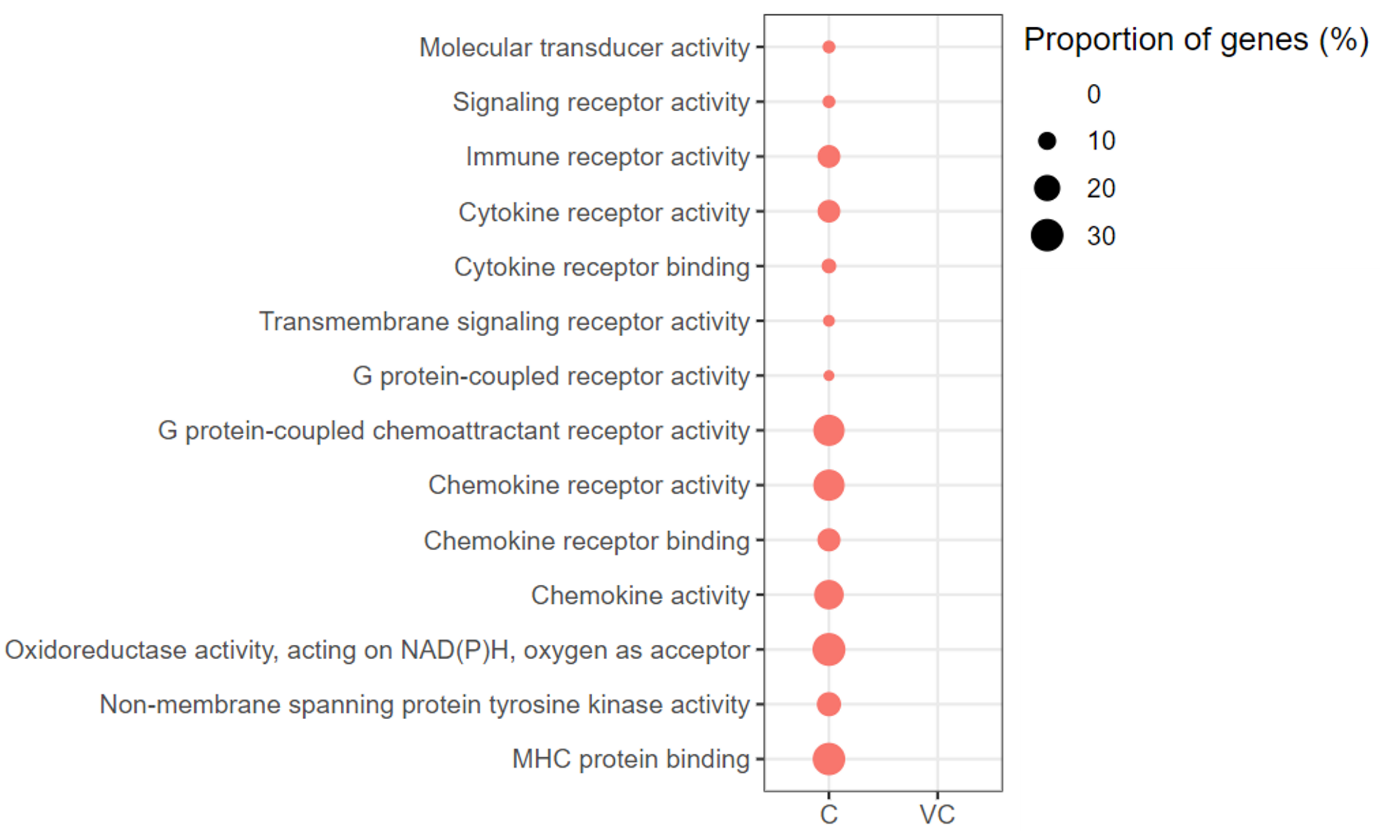

Supplement: Supplementary file 2 — Additional file 2. Gene ontology terms in the category of molecular functions enriched with up-regulated genes. The gene ontology terms in the category of molecular functions enriched with up-regulated genes in the unvaccinated-challenged and vaccinated-challenged chickens, compared to the unvaccinated-unchallenged chickens. [file 13567_2025_1571_MOESM2_ESM.docx]

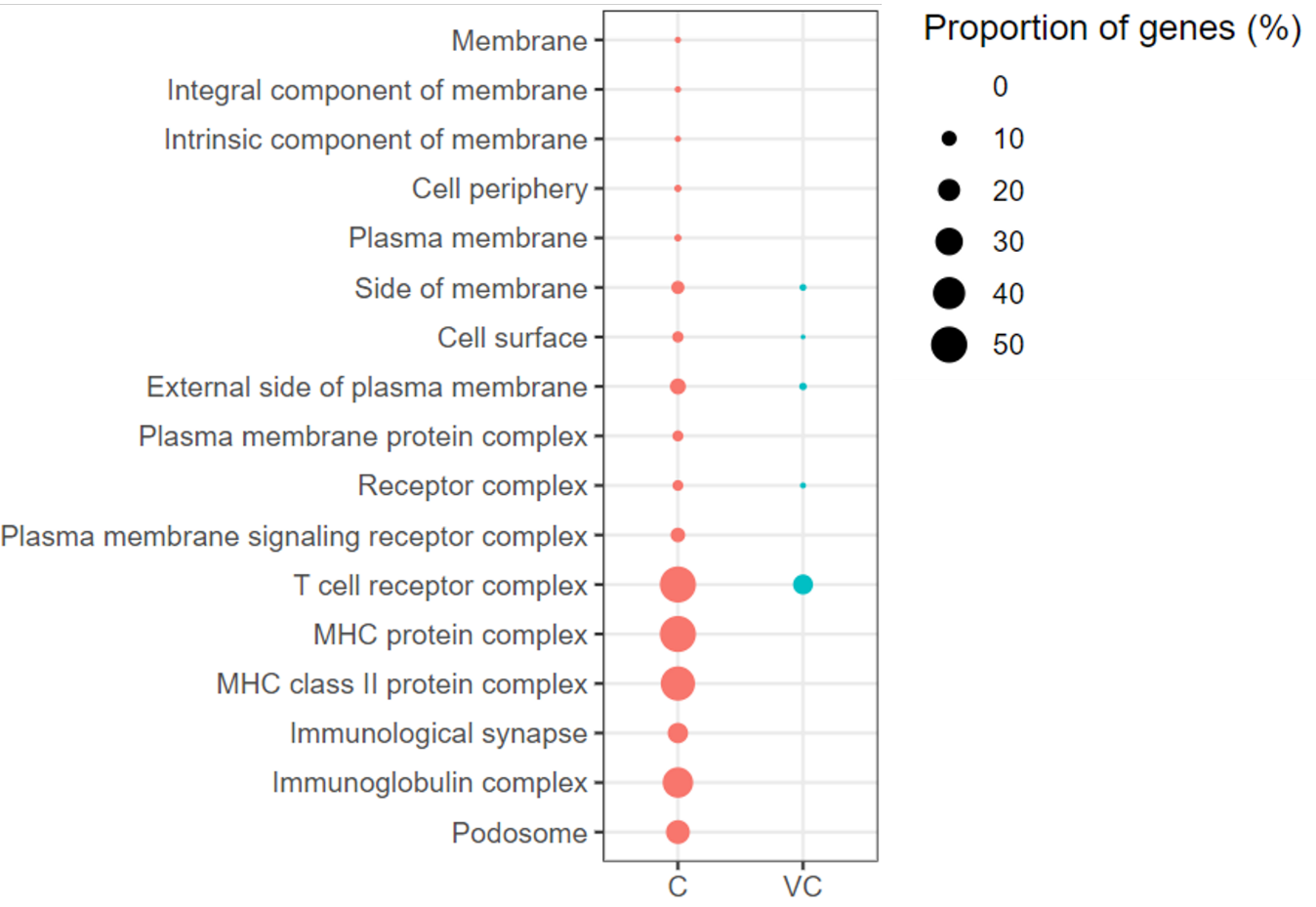

Supplement: Supplementary file 3 — Additional file 3. Gene ontology terms in the category of cellular components enriched with up-regulated genes. The gene ontology terms in the category of cellular components enriched with up-regulated genes in the unvaccinated-challenged and vaccinated-challenged chickens, compared to the unvaccinated-unchallenged chickens. [file 13567_2025_1571_MOESM3_ESM.docx]
