# Supplementary material for: Mechanisms of vaccine protection in chickens against challenge with virulent Mycoplasma synoviae
Source: Vet Res. 2025 Jul 9;56:146. doi: 10.1186/s13567-025-01571-3 (PMC12243254; doi:10.1186/s13567-025-01571-3)
Supplement: Supplementary file 4 — Additional file 4. Down-regulated genes in the vaccinated-challenged chickens. Down-regulated genes in the vaccinated-challenged chickens compared to the unvaccinated-unchallenged chickens. [file 13567_2025_1571_MOESM4_ESM.docx]

**Additional file 4 Down-regulated genes in the vaccinated-challenged chickens compared to the unvaccinated-unchallenged chickens**

| **Gene symbol** | **Gene name** | **Vaccinated-challenged birds** | | **Unvaccinated-challenged birds** | |
| --- | --- | --- | --- | --- | --- |
|  |  | **LFC** | **FDR** | **LFC** | **FDR** |
| **Signal transduction** | | | | | |
| *HTR1D* | 5-hydroxytryptamine (serotonin) receptor 1D, G protein-coupled | -4.14 | 4.12E-05 | -3.53 | 1.04E-04 |
| *RGS2* | regulator of G-protein signaling 2 | -2.14 | 3.58E-03 | -2.24 | 5.77E-04 |
| *PENK* | proenkephalin | -3.50 | 4.12E-02 | -3.61 | 8.95E-03 |
| *WNT9A* | Wnt family member 9A | -2.39 | 4.12E-02 |  |  |
| **Transcription** | | | | | |
| *NR4A3* | nuclear receptor subfamily 4 group A member 3 | -2.61 | 4.12E-02 |  |  |
| *ID2* | inhibitor of DNA binding 2, HLH protein | -1.62 | 4.77E-02 | -1.54 | 3.06E-02 |
| *NOCT* | nocturnin | -1.58 | 4.92E-02 | -1.76 | 2.72E-03 |
| **Immune response** | | | | | |
| *ORM1* | orosomucoid 1 (ovoglycoprotein) | -5.35 | 4.40E-04 | -4.54 | 7.03E-04 |
| *LYZ* | lysozyme (renal amyloidosis) | -1.98 | 2.08E-02 | -1.82 | 1.40E-02 |
| *CCL20* | C-C motif chemokine ligand 20 | -2.75 | 3.39E-02 | -2.86 | 5.99E-03 |
| **Cell adhesion and extracellular matrix associated** | |  |  |  |  |
| *NRCAM* | neuronal cell adhesion molecule | -3.42 | 3.58E-03 | -3.37 | 1.45E-03 |
| *KIRREL3* | kin of IRRE like 3 (Drosophila) | -2.41 | 1.99E-02 | -2.26 | 6.52E-03 |
| *HAS2* | hyaluronan synthase 2 | -2.39 | 4.98E-02 | -2.36 | 1.26E-02 |
| **Metabolism** | | | | | |
| *SLC27A6* | solute carrier family 27 (fatty acid transporter), member 6 | -2.72 | 5.18E-03 | -2.43 | 4.49E-03 |
| *PDK4* | pyruvate dehydrogenase kinase 4 | -2.31 | 4.12E-02 |  |  |
| **Other** | | | | | |
| *TOB1* | transducer of ERBB2, 1 | -2.25 | 2.26E-03 | -2.30 | 5.64E-04 |
| *KCNIP4* | potassium voltage-gated channel interacting protein 4 | -3.08 | 1.06E-02 | -2.98 | 2.75E-03 |
| *CAT* | catalase | -1.86 | 4.12E-02 | -1.84 | 1.23E-02 |
| *FGB* | fibrinogen beta chain | -3.65 | 4.12E-02 | -4.04 | 5.31E-03 |
| *CRISP3* | cysteine rich secretory protein 2 | -8.27 | 4.12E-02 | -9.01 | 6.83E-03 |
| *MBL2* | mannose binding lectin 2 | -2.86 | 4.12E-02 | -2.60 | 1.67E-02 |

LFC = log_2_ fold-change; FDR = false discovery rate (< 0.05 was considered significant)
